# The effectiveness of glucagon-like peptide-1 receptor agonists (GLP-1RAs) on binge eating in patients with obesity: a systematic review and meta-analysis

**DOI:** 10.1016/j.eclinm.2026.104007

**Published:** 2026-07-15

**Authors:** Isobel Emptage, Stella Kozmér, Antemeka Cobham-Wilson, Bernardo Sousa, Kirsty Sugden, Lauren Wheeler, Clare Llewellyn, Janine Makaronidis, Cynthia Bulik, Francesca Solmi, Ilaria Costantini

**Affiliations:** a Division of Psychiatry, University College London, London, United Kingdom; b Department of Health and Community Sciences, Medical School, University of Exeter, Exeter, United Kingdom; c Nuffield Department of Primary Care Health Sciences, University of Oxford, Oxford, United Kingdom; d Department of Behavioural Science and Health, University College London, London, United Kingdom; e Centre for Obesity Research, Division of Medicine, University College London, London, United Kingdom; f NIHR University College London Hospital Biomedical Research Centre, London, UK; g Department of Diabetes and Metabolism, Barts Health NHS Trust, London, UK; h Blizard Institute, Queen Mary University of London, UK; i Department of Medical Epidemiology and Biostatistics, Karolinska Institute, Stockholm, Sweden; j Department of Psychiatry, University of North Carolina at Chapel Hill, Chapel Hill, USA; k Department of Nutrition, University of North Carolina at Chapel Hill, Chapel Hill, USA

**Keywords:** GLP-1 receptor agonists, Binge eating disorder, Obesity, Randomised controlled trials, Eating behaviour, Systematic review and meta-analysis

## Abstract

**Background:**

Glucagon-like peptide-1 receptor agonists (GLP-1RAs), widely used for weight management and type 2 diabetes, may reduce binge eating behaviours and body dissatisfaction, but there are concerns that they might increase eating restraint. We evaluated the effectiveness of GLP-1RAs for binge eating severity and related eating behaviours.

**Methods:**

We conducted a systematic review and meta-analysis of randomised controlled trials (RCTs) comparing any GLP-1RA with placebo or active comparators reporting validated measures of binge eating, disinhibited eating, and loss of control eating. We searched MEDLINE, Embase, PsycINFO, and the Cochrane Central Register of Controlled Trials for RCTs published from Jan 2005 to April 2026. Trials including participants with severe physical comorbidities (e.g., cancer) were excluded. Standardised mean differences (Hedges’ *g*) based on change-from-baseline scores or post-intervention raw mean differences (*MD*) with 95% CIs were estimated using random-effects models, with heterogeneity quantified using I^2^ and τ^2^; publication bias was not formally assessed due to the limited number of studies. Intervention effects on body image concerns were not meta-analysed due to limited data. PROSPERO: CRD42024570728.

**Findings:**

Twenty five RCTs (n = 8069; ∼two-thirds female; majority White) met inclusion criteria. Compared with control conditions, participants allocated to GLP-1RAs reported moderate reductions in binge eating (n = 2106; k = 9; *g* = −0.23 [95% CI −0.36 to −0.10]), loss of control eating (n = 362; k = 5; *MD* = −18.38 [–23.34 to −13.43]) and eating disinhibition (n = 911; k = 13; *g* = −0.54 [−0.90 to −0.18]); alongside lower emotional eating (n = 429; k = 4; *g* = −0.30 [−0.51 to −0.09]) and higher cognitive or dietary restraint (n = 911; k = 13; *g* = 0.31 [0.17–0.44]). Effects were larger in trials enrolling participants with binge eating disorder. All studies were rated as having high risk or some concerns of bias, and heterogeneity was substantial.

**Interpretation:**

GLP-1RAs reduced binge eating-related behaviours and emotional eating and increased cognitive or dietary restraint, though whether restraint is pathological is unclear. Given heterogeneity and high risk of bias, adequately powered trials targeting individuals with diagnosed binge eating disorder and longer follow-up are needed to inform clinical practice.

**Funding:**

Wellcome Trust and Medical Research Foundation.

**Research in context**

**Evidence before this study**

We searched PubMed on January, 2026, using the terms ((“glucagon-like peptide 1 receptor agonist”[Mesh] OR “glucagon-like peptide 1”[Mesh] OR GLP-1RA∗[tiab] OR GLP1RA∗[tiab] OR semaglutide[tiab] OR liraglutide[tiab] OR dulaglutide[tiab] OR exenatide[tiab] OR lixisenatide[tiab] OR tirzepatide[tiab] OR retatrutide[tiab] OR cagrilintide[tiab]) AND (“binge eating”[Mesh] OR “binge eating disorder”[Mesh] OR “bulimia nervosa”[Mesh] OR “eating disorders”[Mesh] OR “eating behavior”[Mesh] OR “feeding behavior”[Mesh] OR “hyperphagia”[Mesh] OR “binge eating”[tiab] OR “binge-eating”[tiab] OR “binge eating disorder”[tiab] OR “loss of control eating”[tiab] OR “disinhibited eating”[tiab] OR “overeating”[tiab])) AND ((systematic[sb]) OR “systematic review”[tiab] OR “meta-analysis”[pt] OR “meta-analysis”[tiab] OR “meta analysis”[tiab]). The search identified 15 reviews. Of these, three directly examined eating-disorder–related outcomes in the context of glucagon-like peptide 1 receptor agonist (GLP-1RAs). Two were narrative reviews that included a small number of early pilot trials (typically fewer than five studies) and did not perform quantitative synthesis, limiting inference about effect magnitude or consistency. Only one review included a meta-analysis, which quantitatively examined changes in emotional eating and dietary restraint, but did not evaluate core binge eating outcomes such as binge eating frequency, binge eating severity, or loss of control eating. No previous meta-analysis systematically assessed the effectiveness of GLP-1RAs on binge eating or loss of control eating as primary outcomes across randomised controlled trials (RCTs).

**Added value of this study**

To our knowledge, this is the most comprehensive synthesis of RCTs evaluating the effectiveness of GLP-1RAs for binge eating–related symptoms. We show consistent reductions in binge eating severity, disinhibited eating, and loss of control eating, suggesting that these agents may have beneficial effects on binge eating in individuals living obesity beyond weight loss. A lack of long-term follow-up data, together with the limited inclusion of participants formally diagnosed with binge eating disorder, limits the generalisability of these findings to sustained treatment effects, potential symptom rebound after treatment discontinuation, and clinically meaningful reductions in binge eating severity.

**Implications of all the available evidence**

For patients living with obesity and/or type 2 diabetes GLP-1RAs may represent a promising adjunctive option for reducing binge eating–related symptoms, but current evidence is preliminary. Most included trials were at high risk of bias, funded by the pharmaceutical companies, and rarely enrolled participants with a clinical diagnosis of binge eating disorder, which substantially limits the certainty and generalisability of the findings. Robust, independently funded RCTs—including those recruiting individuals with diagnosed binge eating disorder, incorporating longer follow-up, and measuring both psychological and metabolic outcomes—are needed to clarify the potential clinical role of GLP-1RAs and determine whether the observed short-term benefits translate into meaningful and sustained improvements.

## Introduction

Binge eating disorder—characterised by recurrent episodes of consuming large quantities of food while experiencing a sense of loss of control—affects over 17 million people worldwide^1^ and is associated with substantial psychiatric and physical morbidity,^2^, ^3^, ^4^ including metabolic complications^5^ and obesity.^6^ Around two-thirds of individuals with binge eating disorder live with overweight or obesity,^7^ and binge eating is common among people seeking weight-loss treatment.^8^ Despite its prevalence and impact, current clinical guidance offers only a limited number of psychological or psychoeducational interventions, whose effects decline over time,^9^ and little to no approved pharmacological treatment options.^10^^,^^11^

Drugs targeting appetite-regulating hormones, particularly glucagon-like peptide-1 receptor agonists (GLP-1RAs)—originally developed for type 2 diabetes and now widely used for weight management—are gaining increasing attention as potential adjunct treatments for reducing binge eating and loss of control eating in people also living with obesity^12^^,^^13^ through both biological and affective pathways. Biologically, GLP-1RAs suppress appetite through direct anorectic effect on the central nervous system, enhance insulin secretion, and delay gastric emptying,^14^ which may reduce uncontrolled food intake. Beyond these effects, GLP-1RAs also cross the blood–brain barrier and may influence affective processes; preclinical and neuroimaging studies suggest that they modulate reward- and impulsivity-related circuits implicated in food cravings and loss of control eating.^15^^,^^16^ Despite these plausible mechanisms, robust causal evidence for their effectiveness in reducing binge eating is lacking.

The few existing systematic reviews and meta-analyses suggest that GLP-1RAs, such as liraglutide, may reduce binge eating symptoms and related behaviours, including emotional eating and difficulties controlling eating.^12^^,^^13^^,^^17^, ^18^, ^19^, ^20^, ^21^, ^22^ However, most relied primarily on narrative synthesis and included only a few pilot trials.^13^^,^^18^^,^^22^^,^^23^ Others combined observational, interventional, and preclinical evidence, which limits causal inference and their relevance for decision-making in human populations.^20^^,^^22^^,^^24^ Even where meta-analyses were conducted,^17^^,^^19^^,^^24^ one included only a single GLP-1RA trial,^17^ another pooled three studies and focused on emotional and restrictive eating rather than binge eating and loss of control eating,^19^ and a third included three GLP-1RA studies while combining retrospective and randomised designs.^24^ Lastly, although observational studies have not reported worsening of eating disorder symptoms among people with obesity prescribed GLP-1RAs,^25^ previous reviews have not explored potential adverse effects. These include increases in restraint, a multidimensional construct encompassing cognitive efforts to limit intake (i.e., cognitive restraint) and behavioural attempts to limit food intake (i.e., dietary restraint),^26^ which may become maladaptive in binge eating disorder,^27^^,^^28^ as well as body dissatisfaction which, albeit not diagnostic, is common in this population.^29^ While body dissatisfaction may improve short term with weight loss, it could also worsen over time through shifts in body ideals and reinforcement of weight-related stigma.^30^

These limitations are particularly important given the rapid expansion of GLP-1RA use in primary care,^31^ where binge eating disorder is often under-recognised, alongside barriers to accessing these medications driven by high cost and stringent eligibility criteria in health care settings, resulting in the exclusion of some individuals with known eating disorders under current guidance.^32^

This systematic review and meta-analysis comprehensively evaluates the effectiveness of GLP-1RAs in reducing or preventing binge eating symptoms and related behaviours and investigates their effects on restrictive-eating behaviours and body image concerns. We also explore whether effects vary by socio-demographic, clinical, and treatment-related characteristics to identify groups more likely to benefit from these medications.

## Methods

### Search strategy and selection criteria

We followed the PRISMA guidelines for systematic review and meta-analysis^33^ and pre-registered our protocol on PROSPERO (CRD42024570728) (See Supplementary Table S1 for PRISMA checklist).

We systematically searched MEDLINE, EMBASE, PsycINFO, and the Cochrane Central Register of Controlled Trials (CENTRAL) via Ovid for trials published from 2005, when GLP-1RAs were first approved, to July 2025 with no language or publication type restrictions. Our search was first conducted on 30th May 2024 and updated in January, July 2025, and April 2026. We also searched *ClinicalTrials.gov* for completed and ongoing studies and manually checked reference lists of included articles and relevant reviews. We contacted authors where relevant. A detailed description of the methods, including search terms, are reported in Appendix pp. 3–6 and Supplementary Table S2.

### Eligibility criteria

We included RCTs evaluating any GLP-1RA or dual/triple agonist versus any comparator (placebo, active drug, or usual care) in participants of any age or diabetic status, if they reported quantitative changes in binge eating symptoms (i.e., primary outcomes) assessed using validated instruments. We excluded studies involving participants with major medical or neurological conditions likely to affect comparability across studies (e.g., cancer, Alzheimer's disease; full list in Supplementary Table S3).

### Study selection

Search results were imported into EndNote 21,^34^ and duplicates removed. Titles and abstracts were screened independently by two reviewers (IC, IE, FS, or SK) using Rayyan.^35^ Disagreements were resolved through discussion or consultation with a senior reviewer (IC or FS).

### Data extraction

For each included trial, one reviewer (IC, IE) extracted data on study characteristics, sample demographics, intervention and comparator details, and outcome characteristics, including follow-up duration, scales, and effect estimates of intention-to-treat (ITT) analyses. A second reviewer (IC, IE, or FS) verified data accuracy.

When more than one dose was available, we extracted the estimate for the maximum dose as our aim was to capture the maximum possible treatment effect. When multiple intervention or control arms were reported, we selected either the most recently approved drug (e.g., tirzepatide over semaglutide) or the most commonly used comparator to maximise comparability across studies. Only one intervention–control comparison per study was included in each meta-analysis, based on these prespecified selection criteria.

When required numerical data were unavailable, we contacted corresponding authors; where values were presented only in graphical format and authors did not provide numerical results,^36^, ^37^, ^38^ we extracted values using *WebPlotDigitizer* (version 4.3). We also extracted basic information from trial protocols when available. Further details on the handling of multiplicity, selection of effect estimates, and formulae used are provided in Appendix p. 3 and Supplementary Figure S1.

### Quality assessment of evidence

Two reviewers (IC, IE) appraised independently the quality of included studies using the revised Cochrane Risk-of-Bias 2 (RoB-2) tool,^39^ categorising trials as low risk, some concerns, or high risk of bias. Disagreements were resolved by consensus.

### Outcomes

Primary outcomes were binge eating behaviours and disinhibited or loss of control eating at any time point available, taking the first endpoint available for each study. Secondary outcomes were emotional eating, cognitive or dietary restraint, and body image concerns, which were extracted if reported but were not required for study inclusion. Lower scores indicate improvement for all primary outcomes and emotional eating, whereas for cognitive/dietary restraint, higher scores reflect greater eating restraint. We also created an overall binge eating-symptoms by selecting one primary measure per study according to a prespecified hierarchy, prioritising i) binge eating, ii) loss of control eating, and iii) disinhibited eating (Appendix p. 4 and Supplementary Table S4 for working definitions of outcomes).

### Data analysis

All meta-analyses were conducted in R (version 4.3) using the *metafor* package. Body image outcomes were not meta-analysed because only one trial reported relevant data. Where possible, missing standard deviations (SDs) were derived from reported standard errors (SEs), confidence intervals (CIs), or other summary statistics, and otherwise imputed or estimated using prespecified assumptions (Appendix pp. 3 and 4).^40^

For primary outcomes, effect sizes were expressed as Hedges' *g* (standardised mean differences) derived from mean-change scores, ensuring comparability across studies and constructs. When mean-change scores were not provided, we computed them from arm-level baseline and post-intervention values, with SDs of change estimated using a prespecified baseline-follow-up correlation of 0.5.^41^ Hedges’ g and its variance were then calculated using standard formulae with small-sample correction.

For the combined primary outcome, only trials reporting change-from-baseline estimates for this item were included, allowing standardisation consistent with other outcomes. In a separate loss-of-control–specific meta-analysis, we included trials reporting post-intervention estimates only, synthesising these as raw mean differences on a harmonised 0–100 scale (Appendix pp. 5–7, see Supplementary Table S5 for formulae used).

We conducted separate random-effects meta-analyses for each primary outcome (binge eating, loss of control eating, disinhibited eating) and for each secondary outcome (emotional eating and cognitive restraint) using inverse-variance weighting with restricted maximum likelihood (REML).^42^ Statistical heterogeneity is presented as I^2^ (low <25%, moderate 25–75%, high >75%) and τ^2^. We did not formally assess publication bias or funnel plot asymmetry because the small number of included studies and high heterogeneity would make these methods underpowered and difficult to interpret reliably.^43^

We performed a subset of prespecified moderation analyses because of the limited number of available trials. When ten or more studies contributed data and the moderator was continuous, we conducted random-effects meta-regression; otherwise, we performed subgroup analyses and formally tested for interaction effects. Moderators included age, baseline body mass index (BMI) and waist circumference (mean), sex and ethnicity (proportion of female and White participants), duration of the intervention (weeks) both total and at full dose when titration was used; dose (mg) of subcutaneous injections; baseline binge eating disorder diagnosis (yes/no); and presence or absence of type 1 or type 2 diabetes (yes/no). As a sensitivity analysis, we created a numeric score from RoB 2 domain judgements (five domains coded 0/1/2 for low, some concerns, and high risk respectively and summed, range 0–10); this was used only for meta-regression and is distinct from the RoB 2 overall judgement. Post hoc sensitivity analyses were conducted for the combined primary outcome, including leave-one-out analyses and analyses excluding studies judged at overall high risk of bias. Full details on covariate scoring and model specifications are provided in Appendix p. 4, post hoc models in Appendix pp. 4 and 5.

### Involvement of people with lived experience

People with relevant lived experience (n = 11) provided feedback through two patient and public involvement (PPI) events, *“Weight-loss drugs and binge eating disorder: acceptability, safety, and concerns”*, which informed interpretation and dissemination of these findings and is reported in Appendix pp. 9–14. All participants were reimbursed and acknowledged. Four participants contributed to the interpretation, write-up, and dissemination of the findings and are therefore included as co-authors on the manuscript. One member of the research team also works from a position of lived experience.

### Role of the funding source

The funding source had no role in any aspects of this study.

## Results

### Literature selection and study characteristics

**Fig. 1:**
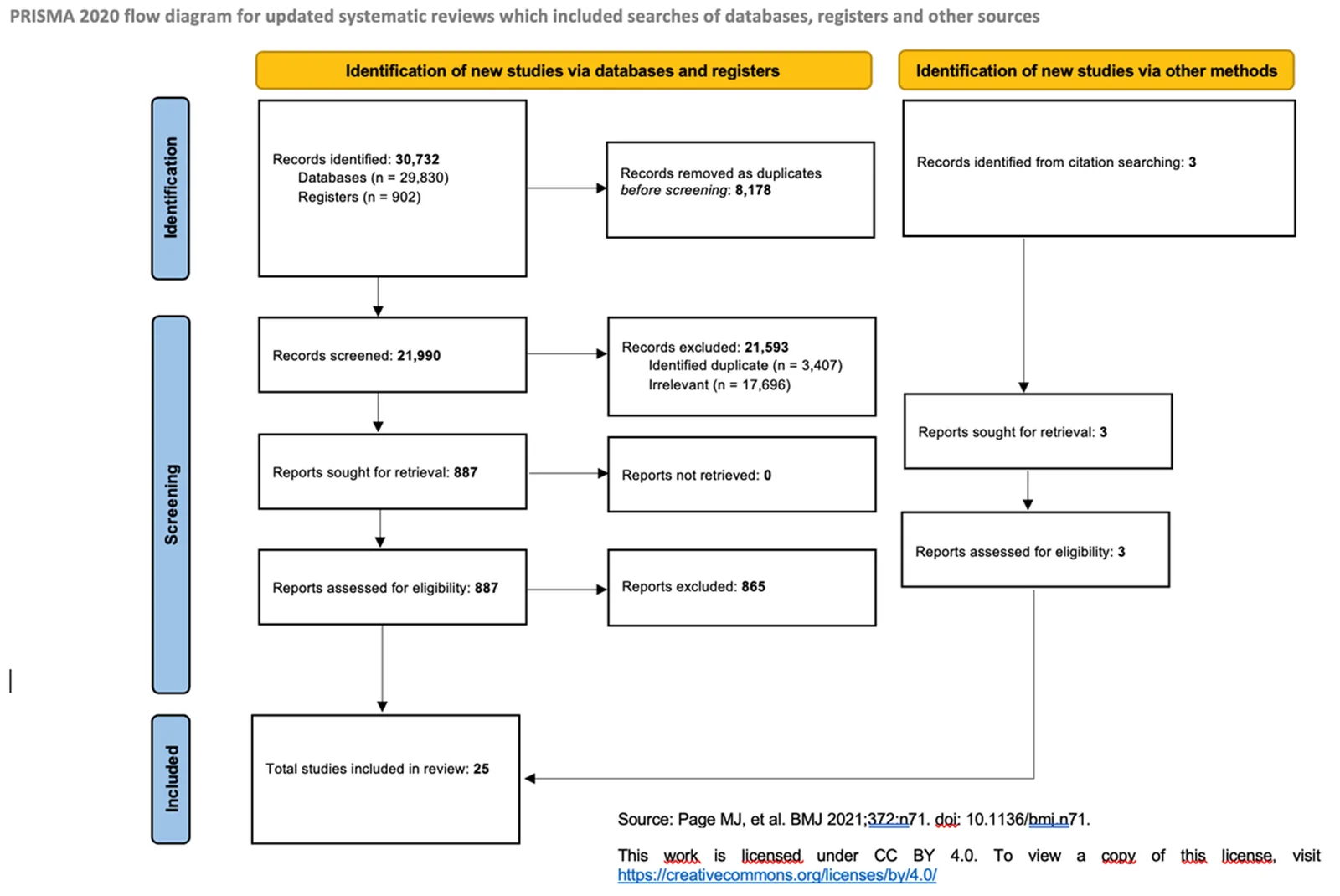
**Flow diagram of study selection.** The search was first run in May 2024 and re-ran in January 2025, July 2025 and April 2026.

Of 30,732 records identified, 887 full-text articles were reviewed and 25 RCTs,^36^, ^37^, ^38^^,^^44^, ^45^, ^46^, ^47^, ^48^, ^49^, ^50^, ^51^, ^52^, ^53^, ^54^, ^55^, ^56^, ^57^, ^58^, ^59^, ^60^, ^61^, ^62^, ^63^, ^64^, ^65^ randomising 8069 participants, met inclusion criteria for the systematic review (Fig. 1 and Table 1).

**Table 1:** Characteristics of studies included in our systematic review.

Author (year)	Study design and country	N randomised	Population demographics	BED and diabetic or other health status	Intervention^a^	Comparator	Outcomes
Allison et al. (2022)	Double-Blind two-arms RCT; United States	37	Age M (SD): 44.2 (10.6) years. Female n (%): 17 (63). White n (%): 16 (59). BMI M (SD): 37.9 (11.8) kg/m^2^.	Individuals meeting full EDE-Q BED criteria^b^ and seeking treatment	3 mg subcutaneous injections of liraglutide once daily for 17 weeks (12 full dose)	Placebo	**Primary:** Post-intervention self-reported binge eating frequency using EDE-Q; eating disinhibition scores using EI; clinician- rated binge eating improvement using CGII **Secondary:** Post-intervention self-reported cognitive restraint scores using EI
Blüher et al. (2024)	Double-Blind eight-arms RCT; United States; Germany	413	Age M (SD): 57.3 (9.8) years. Female n (%): 178 (43.3). White n (%): 344 (83.7). BMI M (SD): 33.9 (6.0) kg/m^2^.	Insulin-naive type 2 diabetics	1 mg semaglutide, 0.3 or 0.9 or 1.8 or 2.7 mg or 1.2 or 1.8 mg subcutaneous survodutide once or twice weekly for 16 weeks **Intervention included in meta-analysis:** Survodutide 2.7 mg	Placebo	**Primary:** Post-intervention changes from baseline self- reported uncontrolled eating scores using TFEQ; difficulty to control eating behaviours using PGI-S **Secondary:** Post- intervention self-reported emotional eating and cognitive restraint scores using TFEQ
Blundell et al. (2017)	Double-Blind crossover (between two arms) RCT; United Kingdom; Denmark	30	Age M (mix max): 42 (21–70) years. Female n (%): 10 (33.3). White n (%): 27 (90). BMI M (min max): 33.8 (30.5–42.8) kg/m^2^.	Non-diabetics	1 mg subcutaneous semaglutide once weekly for 12 weeks (4 full dose)	Placebo	**Primary:** Post- intervention self-reported loss of control eating scores using CoEQ
Chao et al. (2019)	Open-label three-arms RCT; United States	150	Age M (SD): 47.6 (11.8) years. Female n (%): 119 (79.3). White n (%): 81 (54). BMI M (SD): 38.4 (4.9) kg/m^2^.	Non-diabetics with prior unsuccessful weight loss attempts. 52% participants reported at least one binge eating episode in the 28 days prior to treatment	3 mg subcutaneous liraglutide + interpersonal behavioural therapy (IBT) for 52 weeks (48 full dose) or above + 12 weeks low calorie diet **Intervention used in this review:** 3 mg subcutaneous liraglutide + interpersonal behavioural therapy (IBT) for 52 weeks	IBT alone	**Primary:** Post-intervention self-reported binge eating frequency using EDE-Q **Secondary:** Post-intervention self-reported cognitive restraint using EI and dietary restraint scores using EDE-Q
Da Porto et al. (2020)	Open-label two-arms RCT; Outpatient service in Italy	60	Age M (SD): 54.6 (7.7) years. Female n (%): 32 (53.6). White n (%): NI (NI). BMI M (SD): 36.4 (6.2) kg/m^2^.	Type 2 diabetics with a DSM-5 diagnosis of BED	1.5 mg dulaglutide + metformin weekly for 12 weeks	Gliclazide + metformin	**Primary:** Post-intervention self-reported binge eating score change from baseline using BES
Davies et al. (2015)	Double-Blind three-arms RCT; France; Germany; Isreal; South Africa; Spain; Sweden; Turkey; United Kingdom (England and Scotland only); United States	846	Age M (SD): 54.9 (10.5) years. Female n (%): 421 (49.8). White n (%): 705 (83.3). BMI M (SD): 37.2 (6.8) kg/m^2^.	BMI ≥27 plus type 2 diabetes	1.8 mg or 3.0 mg subcutaneous liraglutide for 56 weeks (52 full dose) + lifestyle intervention **Intervention used in this review:** 3.0 mg subcutaneous liraglutide for 56 weeks	Placebo + lifestyle intervention	**Primary:** Post-intervention and short-term self-reported binge eating score change from baseline using BES
Dubé et al. (2020)	Double-Blind crossover (between two arms) RCT; Canada	15	Age M (SD): 35.8 (1.7) years. Female n (%): 8 (53.3). White n (%): NI (NI). BMI M (SD): 30.5 (0.9) kg/m^2^.	Type 1 diabetics	1.8 mg subcutaneous liraglutide + existing insulin treatment for 24 weeks (20 full dose)	Placebo + existing insulin treatment	**Primary:** Post-intervention self-reported eating disinhibition scores using TFEQ **Secondary:** Post- intervention self-reported cognitive restraint scores using TFEQ
Friedrichsen et al. (2020)	Double-Blind two-arms RCT; Germany	72	Age M (SD): 42.8 (11.1) years. Female n (%): 28 (38.9). White n (%): 71 (98.6). BMI M (SD): 34.4 (3.0) kg/m^2^.	Type 1 diabetics. Individuals without dieting attempts in the previous 90 days	2.4 mg subcutaneous semaglutide once weekly for 20 weeks (4 full dose)	Placebo	**Primary:** Post-intervention self-reported control of eating scores change from baseline using CoEQ
Gabe et al. (2024)	Double-Blind two-arms RCT; Germany; United States	61	Age M (SD): 44 (11) years. Female n (%): 21 (34.4). White n (%): 60 (98.4). BMI M (SD): 34.9 (3.8) kg/m^2^.		50 mg oral semaglutide once daily for 20 weeks (4 full dose)	Placebo	**Primary:** Post-intervention self-reported control of eating scores using CoEQ
Gatta-Cherifi et al. (2024)	Double-Blind two-arms RCT; France	41	Age M (SD): 40.4 (13.6) years. Female n (%): 17 (42.5). White n (%): NI. Grade 1 obesity (BMI 30–34.9 kg/m^2^) n (%): 11 (56.5).		10 mcg subcutaneous exenatide twice daily for 26 weeks (22 full dose) + lifestyle intervention	Placebo + lifestyle intervention	**Primary:** Post-intervention self-reported eating disinhibition using TFEQ and EI **Secondary:** Post-intervention self-reported cognitive restraint using TFEQ and EI
Gibbons et al. (2020)	Double-Blind Crossover (between two arms) RCT; United Kingdom	15	Age M (min max): 58.2 (37–73) years. Female n (%): 2 (13.3%). White n (%): 15 (100%). BMI M (min max): 30.8 (26.1–34.6) kg/m^2^.	Type 2 diabetics	14 mg oral semaglutide once daily for 12 weeks, following dose escalation from 3 mg to 7 mg to 14 mg	Placebo/Washout	**Primary:** Post-intervention self-reported control of eating scores using CoEQ
Gudbergsen et al. (2021)	Double-Blind two-arms RCT; Denmark	156	Age M (SD): 59.2 (10.3) years. Female n (%): 101 (65). White n (%): NI (NI). BMI M (SD): 32.1 (4.9) kg/m^2^.	Non-diabetic individuals with symptomatic knee osteoarthritis	3 mg liraglutide for 52 weeks + group dietician sessions for 8 weeks	Placebo for 52 weeks + group dietician sessions for 8 weeks	**Primary:** Post-intervention self-reported binge eating scores using BES
Jensen et al. (2022)	Double-Blind four-arms RCT; Denmark	195	Age M (SD): 45 (12) years. Female n (%): 80 (62). White n (%): NI (NI). BMI M (SD): 36.9 (2.9) kg/m^2^.	Non-diabetics pre-treated with 8 weeks low calorie diet	3 mg liraglutide for 52 weeks or 3 mg liraglutide + exercise for 52 weeks **Intervention used in this review:** 3 mg liraglutide for 52 weeks	Placebo or placebo + exercise **Control used in this review:** Placebo alone	**Primary:** Post- intervention self-reported eating disinhibition scores using TFEQ **Secondary:** Post- intervention self-reported emotional eating and cognitive restraint scores using TFEQ
Jensterle et al. (2023)	Single-Blind; RCT; Slovenia	30	Age M (SD): 33.7 (6.1). Female n (%): 30 (100). White n (%): NI (NI). BMI M (SD): 36.4 (4.4) kg/m^2^.	Obese women with polycystic ovary syndrome	1 mg subcutaneous self-injected semaglutide once weekly for 16 weeks	Placebo	**Primary:** Post- intervention self-reported eating disinhibition scores using TFEQ **Secondary:** Post-intervention self-reported emotional eating and cognitive restraint scores using TFEQ
Kanu et al. (2025) [1]	Double-Blind eight-arms RCT; United States	281	Age M (SD): 56.2 (9.7) years. Female n (%): 156 (56). White n (%): 235 (84). BMI M (SD): 35.0 (6.3) kg/m^2^.	Type 2 diabetics	1.5 mg subcutaneous self-injected dulaglutide once weekly for 36 weeks. Or either 0.5, 2 dose-escalated to 4, 4, 2 dose-escalated to 8, 4 dose-escalated to 8, or 2 dose-escalated to 12 mg subcutaneous self-injected retatrutide once-weekly for 36 weeks **Intervention used in this review:** 12 mg subcutaneous self-injected retatrutide once-weekly for 36 weeks	Placebo	**Primary:** Post-intervention self-reported disinhibition scores using EI **Secondary:** Post-intervention self-reported dietary restraint scores using EI
Kanu et al. (2025)[2]	Double-Blind seven-arms RCT; United States	338	Age M (SD): 48.2 (12.7) years. Female n (%): 163 (48). White n (%): 298 (88). BMI M (SD): 37.3 (5.7) kg/m^2^.	Individuals with obesity or overweight with an obesity-related complication	1, 2 dose-escalated to 4, 4, 2 dose-escalated to 8, 4 dose-escalated to 8, or 2 dose-escalated to 12 mg subcutaneous self-injected retatrutide once-weekly for 48 weeks + diet and physical activity counselling **Intervention used in this review:** 12 mg subcutaneous self-injected retatrutide once weekly for 48 weeks + diet and physical activity counselling	Placebo + diet and physical activity counselling	**Primary:** Post-intervention self-reported disinhibition scores using EI **Secondary:** Post-intervention self-reported cognitive restraint scores using EI
Lau et al. (2021)	Double-Blind seven-arms RCT; Canada; Denmark; Finland; Ireland; Japan; Poland; Serbia; South Africa; United Kingdom; United States	706	Age M (SD): 52.3 (10.6) years. Female n (%): 436 (62). White n (%): 543 (77). BMI M (SD): 37.8 (7.0) kg/m^2^.	Individuals without diabetes	3 mg liraglutide, 0.3, 0.6, 1.2, 2.4, 4.5 mg dose-escalated subcutaneous self-injected cagrilinitide once-weekly for 26 weeks (20 full dose) + dietary and physical activity counselling **Intervention used in this review:** 3 mg subcutaneous self-injected liraglutide once daily for 26 weeks (20 full dose) + dietary and physical activity counselling	Placebo + dietary and physical activity counselling	**Primary:** Post- intervention self-reported uncontrolled eating scores using TFEQ **Secondary:** Post- intervention self-reported emotional eating and cognitive restraint scores using TFEQ
Martin et al. (2024)	Double-Blind/open-label three-arm RCT; United States	114	Age M (SD): 44.9 (10.5). Female n (%): 97 (85). White n (%): 83 (73). BMI M (SD): 36.2 (5.6) kg/m^2^.	Individuals with BMI 27–50 and without diabetes	3 mg dose escalated (1 week full dose) subcutaneous self-injected liraglutide once daily for 6 weeks; 10 mg dose-escalated (2 weeks full dose) subcutaneous self-injected tirzepatide once weekly for 6 weeks **Intervention used in this review:** 10 mg dose escalated (2 weeks full dose) subcutaneous self-injected tirzepatide once weekly for 6 weeks	Placebo	**Primary:** Post- intervention self-reported eating disinhibition scores using EI **Secondary:** Post- intervention self-reported cognitive restraint scores using EI
McElroy et al. (2024)	Double-Blind two-arms RCT; United States	60	Age M (SD): 43.5 (NI) years. Female n (%): 48 (80). White n (%): 55 (91.7). BMI M (SD): 38 (NI) kg/m^2^.	Individuals with stable type 1 or 2 bipolar disorder	3 mg subcutaneous self-injected liraglutide for 40 weeks + nutrition and lifestyle modification counselling	Placebo + nutrition and lifestyle modification counselling	**Primary:** Post- intervention self-reported binge eating scores change from baseline using BES **Secondary:** Post- intervention self-reported cognitive restraint scores using TFEQ
Patino et al. (2025)	Double-Blind two-arms RCT; United States	54	Intervention Age M (SD): 44 (NI) Control Age M (SD): 39 (NI) Female n (%): 36 (66.7) White n (%): 30 (55.6) Intervention BMI M (SD): 31.7 (4.4) Control BMI M (SD): 34.3 (9.2).	Individuals with stable major mood or psychotic disorders	5 mcg dose escalated to 10 mcg subcutaneous self-injected exenatide twice daily for 16 weeks + olanzapine	Placebo + olanzapine	**Primary:** Post-intervention self-reported eating disinhibition scores using TFEQ **Secondary:** Post-intervention self-reported cognitive restraint scores using TFEQ
Pi-Sunyer et al. (2015)	Double-Blind two-arms RCT; United States, Argentina, Australia, Austria, Belgium, Brazil, Canada, Denmark, Finland, Former Serbia and Montenegro, France, Germany, Hong Kong, Hungary, India, Ireland, Israel, Italy, Mexico, Netherlands, Norway, Poland, Russian Federation, Serbia, South Africa, Spain, Switzerland, Turkey, United Kingdom	3731	Age M (SD): 45.1 (12.0). Female n (%): 2928 (78.5). White n (%): 3168 (85%). BMI M (SD): 38.3 (6.3) kg/m^2^.	Individuals with a stable body weight and a BMI of 30 or higher	3 mg liraglutide subcutaneous self-injected once daily for 56 weeks (52 full dose) + lifestyle intervention	Placebo + lifestyle intervention	**Primary:** Post- intervention self-reported binge eating scores change from baseline using BES
Robert et al. (2015)	Open-label two-arms RCT; Malaysia	44	Age M (SD): 34 (9) years. Female n (%): NI (NI). White n (%): NI (NI). BMI M (SD): 35.9 (4.2) kg/m^2^.	Non-diabetic individuals with moderate binge eating^c^	1.8 mg liraglutide plus diet and exercise intervention for 12 weeks	Diet and exercise intervention	**Primary:** Post- intervention self-reported binge eating scores change from baseline using BES
Snaith et al. (2025)	Double-Blind two-arms RCT; Australia	24	Age M (SD): 41 (10) years. Female n (%): 10 (42). White n (%): 22 (91.7). BMI M (SD): 33.7 (3) kg/m^b^.	Individuals with type 1 diabetes	2.5 mg dose-escalated to 5 mg subcutaneous self-injected tirzepatide once-weekly for 12 weeks	Placebo	**Primary:** Post-intervention self-reported uncontrolled eating scores using TFEQ **Secondary:** Post-intervention self-reported cognitive restraint and emotional eating scores using TFEQ
Wadden et al. (2013)	Double-Blind two-arms RCT; United States; Canada	422	Age M (SD): 46.2 (11.5) years. Female n (%): 343 (81.3). White n (%): 355 (84.1). BMI M (SD): 37.9 (6.2) kg/m^2^.	Non-diabetic individuals without an eating disorder diagnosis and with previous unsuccessful weight-loss attempts	3 mg subcutaneous self-injected liraglutide once-daily for 56 weeks (52 full dose) + 12 weeks low calorie diet	Placebo + 12 weeks low calorie diet	**Primary:** Post- intervention self-reported binge eating using BES scores
Wharton et al. (2023)	Double-Blind two-arms RCT; United States; Canada	174	Age M (SD): 47.3 (11.5) years. Female n (%): 135 (77.6). White n (%): 154 (88.5). BMI M (SD): 38.7 (7.4) kg/m^2^.	Self-reported unsuccessful dietary weight- loss attempts, without diabetes	2.4 mg subcutaneous semaglutide once weekly plus lifestyle modification for 104 weeks	Placebo plus lifestyle modification	**Primary:** Post- intervention self-reported control of eating scores using CoEQ

M = Mean, min = minimum, max = maximum, kg/m^2^ = kilogrammes per square metre, DSM-5 = Diagnostic and Statistical Manual of Mental Disorders, Fifth Edition, BMI = Body Mass Index, RCT = Randomised Controlled Trial, SD = Standard Deviation, NI = No Information, NA = Not Applicable, EDE-Q = eating disorder examination questionnaire, TFEQ = three factor eating questionnaire, BES = binge eating scale, BED = binge eating disorder, EI = eating inventory, CGII = clinician global impressions-improvement, CoEQ = control of eating questionnaire, PGI-S = patient's global impression of severity, IBT = intensive behavioural therapy.

a Intervention described by dose, mode of delivery, drug type, frequency, then duration.

b Full EDE-Q BED criteria = aligns with DSM-5 criteria of recurrent episodes of binge eating and presence of associated features.

c Moderate binge eating = BES score above 18.

24 trials provided useable outcome data and were included in the meta-analysis. Eleven ongoing trials were also identified (Appendix p. 30; Supplementary Table S6).

The 25 trials were conducted across 12 countries on four continents, all high- or upper-middle-income settings, with 21 (84.0%) undertaken in Western countries only. Trial sample sizes ranged from 15^51^^,^^53^ to 3731^64^ participants, and intervention durations varied between 6^62^ and 104^66^ weeks.

Of the 8069 participants randomised across included trials, sex was reported for 8025 (99.5%). Among those with reported sex, 5416 (67.5%) were female. Across studies, the median proportion of female participants was 53.6% (range: 13.3%^53^ to 97.0%^56^). Where reported, the proportion of White participants was high (median: 84.7%; range: 54.0%^48^ to 100%^46^^,^^53^).

The median mean age across studies was 47.3 years (Interquartile Range, IQR: 43.0–54.9; k = 25). All studies recruited participants living with overweight or obesity. The median baseline mean BMI was 35.8 kg/m^2^ (IQR: 33.8–37.4; k = 22), and the median baseline mean waist circumference was 113.0 cm (IQR: 108.5–115.3; k = 17).

Ten trials included participants with diabetes (type 1 or type 2)^38^^,^^44^^,^^45^^,^^49^, ^50^, ^51^^,^^53^^,^^59^^,^^64^ or pre-diabetes.^60^ Only three trials actively recruited participants with a diagnosis of binge eating disorder or identified as binge eaters to specifically test the effectiveness of GLP-1RAs on binge eating.^47^^,^^49^^,^^61^

Liraglutide was the most frequently investigated GLP-1RA, examined in 12 trials (48.0%), followed by semaglutide in six trials (24.0%), retatrutide, dulaglutide, exenatide and tirzepatide in two trials each (8.0%), and survodutide and cagrilinitide in one trial each (4.0%) (Table 1 and Supplementary Tables S7 and S8). Six trials (24.0%) included multiple active intervention arms, and three (12.0%) evaluated more than one GLP-1–based compound within the same study. Doses varied according to compound and formulation, ranging from 0.01 mg^44^ to 12 mg^37^ for injectable agents administered subcutaneously once daily or once weekly, and from 14 mg^53^ to 50 mg^36^ for oral formulations. All but one^49^ used stepwise dose-escalation schedules (i.e., titration) to improve tolerability, and 88.0% used placebo as the main comparator,^36^, ^37^, ^38^^,^^45^^,^^47^^,^^50^^,^^52^^,^^54^^,^^56^, ^57^, ^58^, ^59^, ^60^^,^^62^, ^63^, ^64^, ^65^ with the remaining studies using the second-line type 2 diabetes treatment gliclazide plus metformin,^49^ diet and exercise modification,^61^ or intensive behavioural therapy.^48^

**Fig. 2:**
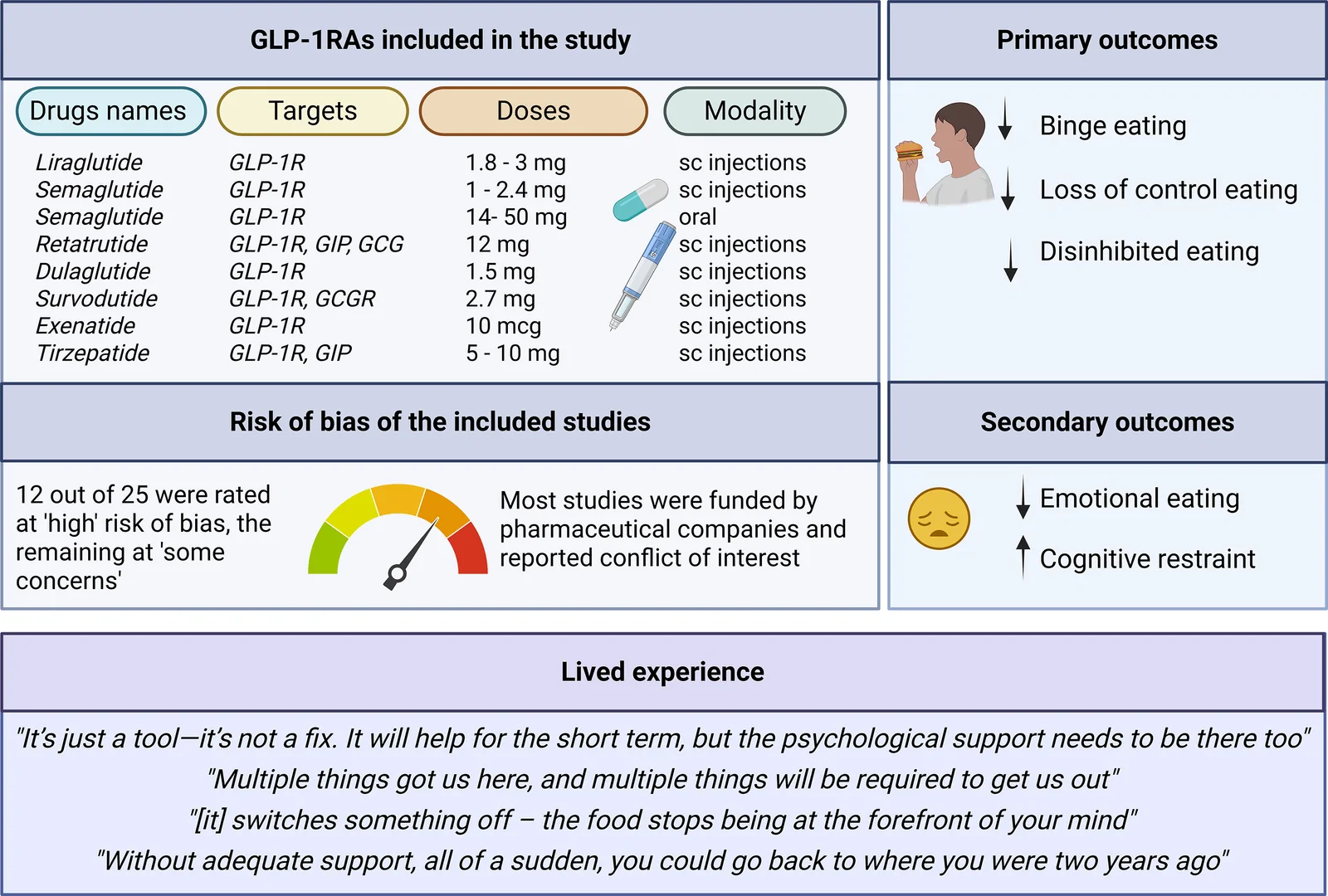
**Visual summary of included studies and main findings.** GLP-1RAs = glucagon-like peptide-1 receptor agonists; GLP-1R = glucagon-like peptide-1 receptor; GIP = glucose-dependent insulinotropic polypeptide; GCG = glucagon; GCGR = glucagon receptor; sc = subcutaneous; mcg = micrograms; mg = milligrammes; PPI = Patient and Public Involvement.

Studies most commonly assessed binge eating symptoms using the Binge Eating Scale (BES),^67^ and measured eating disinhibition—as well as the secondary outcomes cognitive restraint and emotional eating—using the Three-Factor Eating Questionnaire (TFEQ).^66^ Studies assessed loss of control eating using item 19 of the Control of Eating Questionnaire (CoEQ).^68^ Details of all reported scales are provided in Appendix p. 7. A summary of main study characteristics and findings is illustrated in Fig. 2.

### Risk of Bias (RoB)

**Fig. 3:**
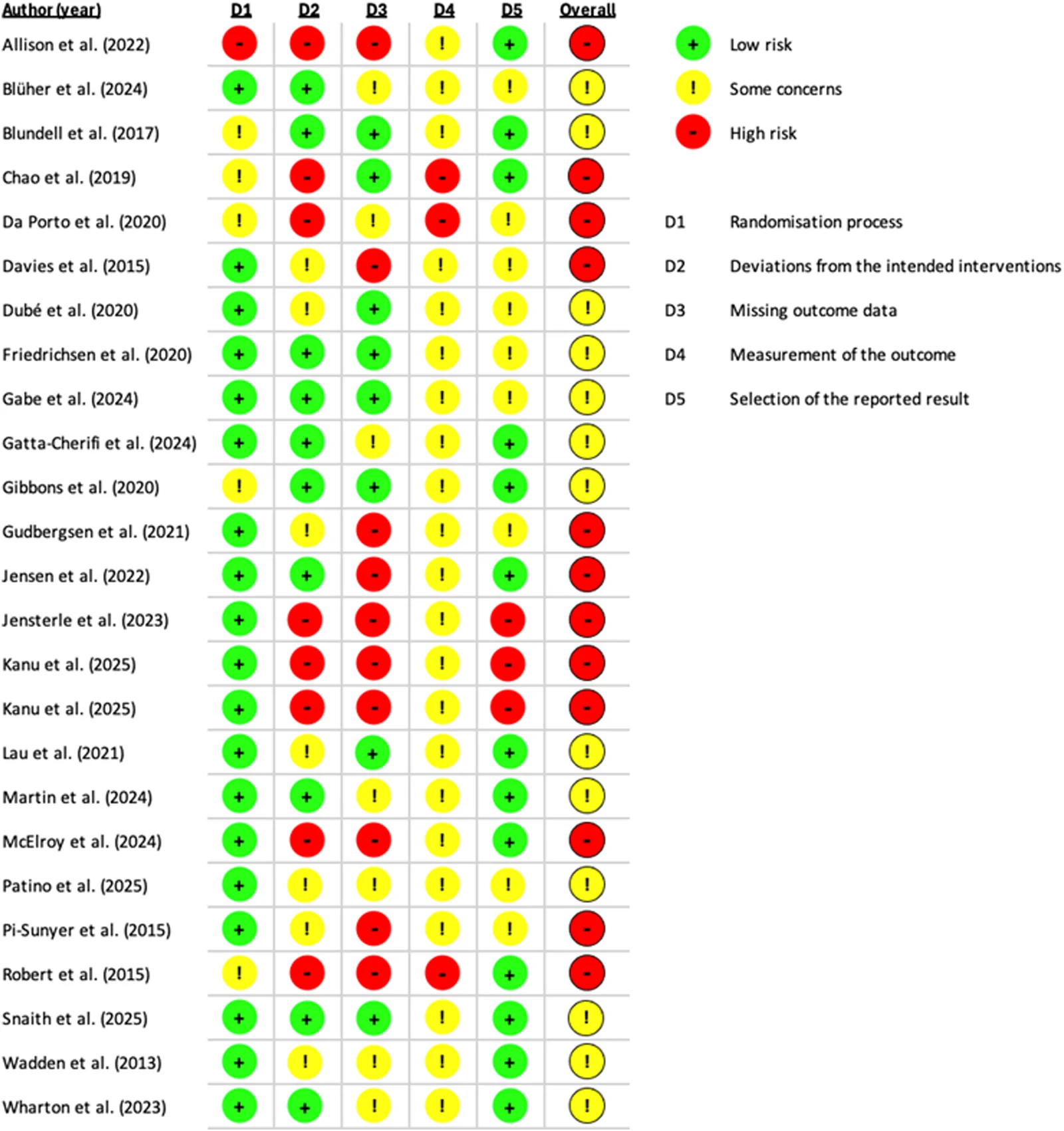
**A traffic light plot to show the risk of bias in each risk of bias (RoB 2.0) domain**.

Across the 25 trials assessed with the Cochrane ROB-2 tool, we rated 12 trials as having high overall risk of bias, with the remaining rated as having some concerns. The domains most frequently judged at high risk were deviations from intended interventions and missing outcome data. These ratings were driven by treatment mis-dispensing, post-randomisation exclusions, differential attrition. In contrast, we rated the randomisation process as low risk in most trials. We considered outcome measurement and selective reporting as presenting some concerns, because treatment effects could lead to possible functional unblinding (see Appendix p. 7 for additional details on RoB rating by domain, and Fig. 3 and Supplementary Table S9).

### Primary outcomes

**Fig. 4:**
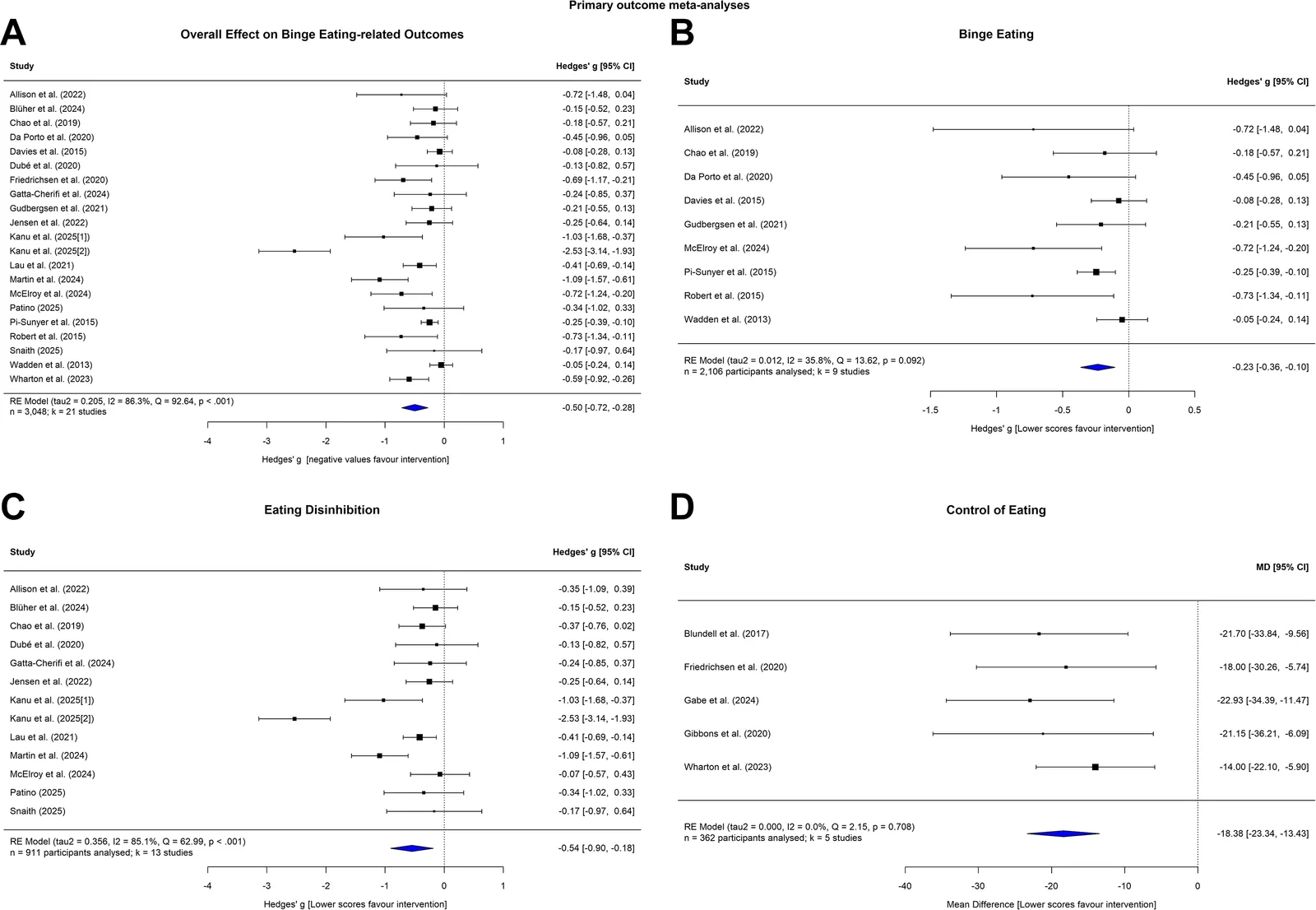
**Forest plots illustrating the effects of GLP-1RAs on primary outcomes.(A) Combined primary outcome (all binge eating–related outcomes). (B) Binge eating. (C) Eating disinhibition. (D) Control of eating.** Effect estimates are standardised mean differences (Hedges' g) with 95% confidence intervals (CIs) for binge eating, eating disinhibition, and the combined primary outcome, where negative values indicate greater improvement with glucagon-like peptide-1 receptor agonists (GLP-1RAs) compared with control. These estimates are based on change from baseline. For loss of control eating, effects are reported as mean differences (intervention − control) in post-intervention Control of Eating Questionnaire (CoEQ) item-19 scores on the harmonised 0–100 scale. The pooled estimate (blue diamond) reflects the average treatment effect; n = number of participants; k = number of studies; τ^2^ quantifies between-study variance, and I^2^ reflects the proportion of variability attributable to heterogeneity rather than sampling error.

Participants randomised to a GLP-1RA intervention showed consistent reductions in binge eating-related symptoms compared with controls, with small-to-moderate reductions in binge eating severity (Hedges’ *g* = −0.23 [−0.36 to −0.10], n = 2106; k = 9), disinhibited eating (*g* = −0.54 [−0.90 to −0.18], n = 911; k = 13), and loss of control eating (*MD* = −18.38 [−23.34 to −13.43], n = 362; k = 5). When binge eating–related outcomes were combined, GLP-1RA treatment remained associated with a moderate overall reduction in symptom severity (*g* = −0.50 [−0.72 to −0.28], n = 3048; k = 21) (Fig. 4). Heterogeneity was high for pooled and eating disinhibition analyses but moderate to low for loss of control and binge eating, suggesting consistent treatment effects across studies.

### Secondary outcomes

**Fig. 5:**
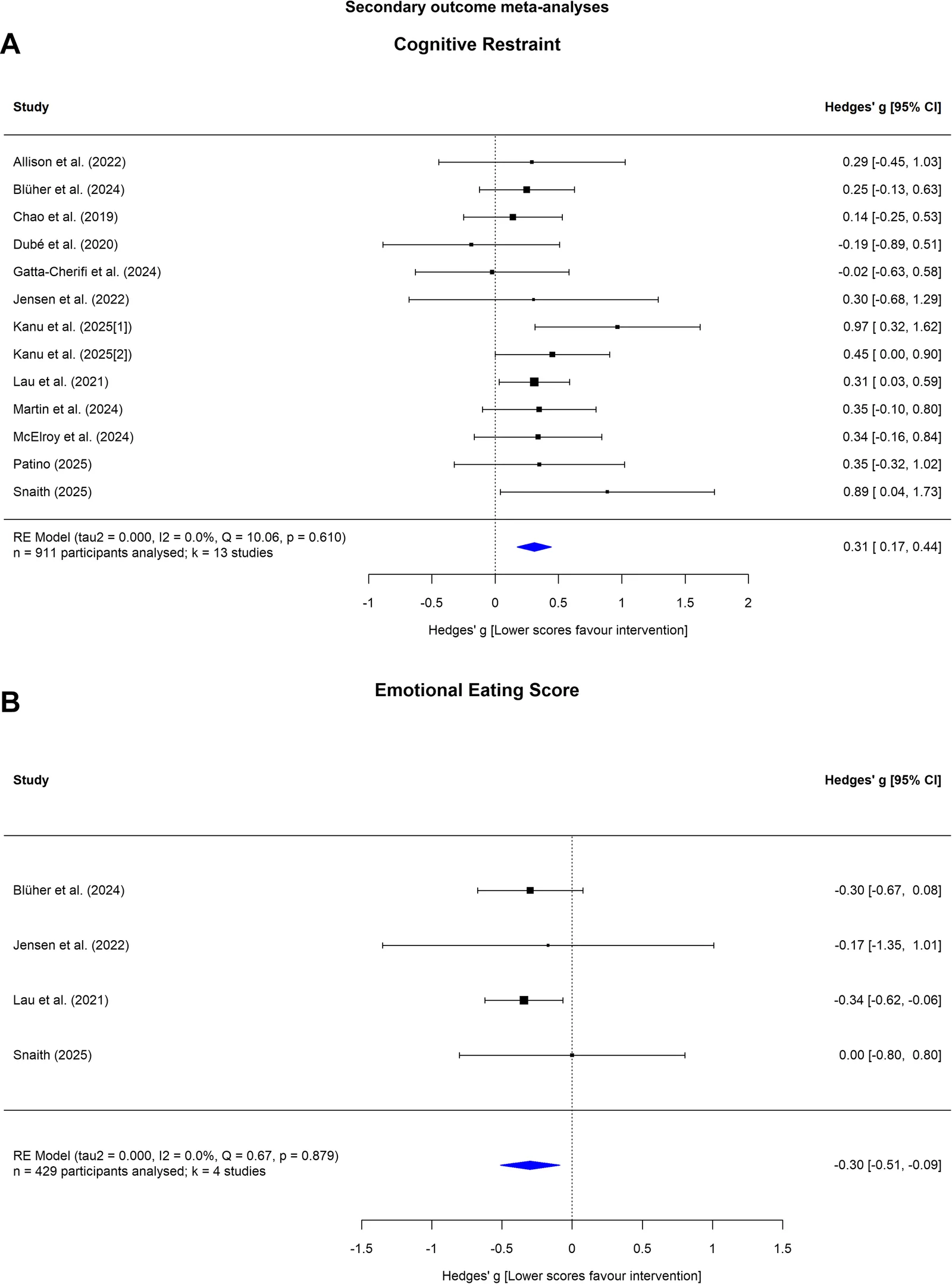
**Forest plots illustrating the effects of GLP-1RAs on secondary outcomes.(A) Cognitive restraint. (B) Emotional eating.** Effect estimates are standardised mean differences (Hedges' g) with 95% confidence intervals (CIs), where negative values indicate greater improvement with glucagon-like peptide-1 receptor agonists (GLP-1RAs) compared with control for emotional eating whereas higher values for the cognitive restraint outcome suggest greater cognitive or dietary restraint. The pooled estimate (blue diamond) reflects the average treatment effect; n = number of participants; k = number of studies; τ^2^ quantifies between-study variance, and I^2^ reflects the proportion of variability attributable to heterogeneity rather than sampling error.

Participants receiving GLP-1RAs reported moderate reduction in emotional eating scores compared with controls (*g* = −0.30 [95% CI −0.51 to −0.09], n = 429; k = 4), although precision was limited by the small number of studies. GLP-1RA treatment was also associated with a moderate increase in cognitive and dietary restraint (*g* = 0.31 [0.17–0.44], n = 911; k = 13) (Fig. 5). Heterogeneity was negligible for both emotional eating and cognitive restraint, indicating consistent direction of effects.

### Subgroup and sensitivity analyses

We did not find strong evidence that any of the continuous study- or participant-level characteristics moderated the intervention effects (Supplementary Figure S2), except for trials that included participants with baseline binge eating disorder and/or directly targeted binge eating where the effect sizes were larger (*g* = −0.60 [95% CI −0.95 to −0.25], n = 129, k = 3) compared with trials that did not (*g* = −0.27 [−0.39 to −0.15], n = 2628, k = 14; interaction term: −0.33 [−0.73 to 0.06], p = 0.10) (Supplementary Figures S3 and S4). Across all models, τ^2^ and I^2^ remained high.

Post hoc leave-one-out analyses and sensitivity analyses excluding studies at overall high risk of bias were consistent with the main findings (Appendix pp. 6 and 7, Supplementary Figures S5 and S6).

## Discussion

Our systematic review and meta-analysis provide timely and much-needed evidence that GLP-1RAs reduce binge eating symptoms and associated behaviours, including loss of control eating and disinhibited eating, as well as emotional eating, in individuals living with overweight or obesity. Although we could not explore effects on body image concerns due to limited data, we found evidence for an increase in cognitive or dietary restraint, which may reflect either more flexible, adaptive control or more rigid restraint associated with disinhibition and binge eating.^69^

We did not find strong evidence to support effect moderation across participant and intervention characteristics in subgroup and meta-regression analyses, but we found little evidence that the intervention was more effective in individuals with diagnosed binge eating disorder and/or studies that aimed specifically at reducing binge eating.

Most studies were rated at some concerns of risk of bias, and several at high risk of bias. All studies that included patients with binge eating disorder or that aimed at reducing binge eating were rated as at high risk of bias.

To our knowledge, this is the largest and most up-to-date systematic review and meta-analysis evaluating GLP-1RAs in relation to binge eating and related behavioural outcomes.

Several limitations should be considered when interpreting our findings. Despite comprehensive searches across multiple databases and efforts to obtain unpublished data from study authors, only 25 RCTs were eligible for inclusion in the systematic review, of which 24 contributed to the meta-analysis. The relatively modest number of studies, together with variability in outcome measures, follow-up durations, and participant and intervention characteristics, limited the feasibility of more granular subgroup or dose–response analyses and may have reduced our ability to detect true moderation effects. Meta-regression findings should therefore be interpreted cautiously, as these analyses are inherently underpowered when based on a small number of studies. Substantial heterogeneity further reduced the precision and interpretability of pooled estimates, particularly in the combined primary outcome where related but non-equivalent constructs were pooled, limiting comparability across studies. To maximise inclusion, data from a small number of studies were extracted from published figures using *WebPlotDigitizer*, which may have introduced minor imprecision. However, any resulting measurement error is likely to be random and non-differential rather than systematic. In addition, several effect estimates were derived from data provided by authors where reporting was incomplete or inconsistent. These conversions, including the imputation of missing variability measures (e.g., SDs), required assumptions about data distribution and comparability across studies, which may have introduced further uncertainty. However, results were materially unchanged in sensitivity analyses using alternative plausible correlation coefficients.

Most of the included studies investigated liraglutide, one of the first GLP1-RA licenced for use in management of type 2 diabetes. Newer agents, such as semaglutide and tirzepatide have demonstrated greater appetite-suppressing and weight-reducing effects.^70^ If reductions in binge eating are partly mediated through these mechanisms—which we were unable to test—our pooled estimates may underestimate the potential efficacy of newer compounds. In this meta-analysis, we prioritised ITT analyses. Although ITT estimates are the most appropriate for assessing the effect of being randomised to an intervention and reduce bias arising from lack of blinding, they may underestimate the effect of actually receiving the intervention.

Only three trials recruited participants with a formal binge eating disorder diagnosis and/or specifically aimed to test the effectiveness of GLP-1RAs in reducing binge eating, and most participants across included trials were White, middle-aged adults. Individuals with obesity beyond stage four or those who had tried weight-loss treatment previously or experienced different mental health problems were typically excluded. These factors limit the generalisability of our findings to ethnic minority groups, other age ranges and individuals with severe or treatment-resistant obesity and co-occurring psychiatric difficulties, who may be more representative of the broader clinical population who seek weight-loss treatments and experience binge eating disorder.^8^

As most studies assessed short-term effects, evidence on long-term maintenance, plateau, or rebound after discontinuation remains scarce. Given that weight regain and rebound increases in appetite often follows treatment cessation, longer-term trajectories, including relapse in binge eating behaviours, should be further investigated.

Several studies were rated as having a high risk of bias—most commonly due to deviations from intended interventions, missing outcome data, selective reporting, and potential unblinding related to pronounced effects and well-recognised side-effects of medications; yet, results were robust to sensitivity analyses exploring the impact of high-risk studies. Lastly, most studies were conducted or funded by pharmaceutical companies, raising concerns about sponsorship effects and possible overestimation of treatment effects.

Given the low quality of the available evidence, substantial heterogeneity across studies, and the limited inclusion of participants with clinically diagnosed binge eating disorder, there is a clear need for more robust, independently funded, and adequately powered trials. Future studies should prioritise recruiting individuals with clinically diagnosed or subthreshold binge eating disorder and more carefully characterise outcomes related to eating behaviour, including the distinction between adaptive and potentially pathological forms of eating restraint.

In addition, longer-term studies are needed to evaluate the durability of treatment effects and the risk of relapse following discontinuation. For example, existing evidence suggests that individuals experience faster weight regain after cessation of incretin-based pharmacological treatments compared with behavioural interventions^71^; however, the implications of this for binge eating outcomes remain unclear. Future research should also investigate combined treatment approaches to determine whether GLP-1RAs enhance or attenuate the effectiveness of psychological interventions. This is particularly relevant given anecdotal clinical evidence suggesting that GLP-1RAs may interfere with therapeutic work in, for example, cognitive behavioural therapy by reducing symptoms (e.g., food cravings) that are typically the focus of behavioural experiments and skills practice. Furthermore, incorporating mechanistic outcomes could clarify the neurobiological and psychological pathways through which GLP-1RAs influence binge eating, including whether effects are driven primarily by metabolic changes, appetite suppression and weight loss, or by modulation of inhibitory and reward processes in the brain.^72^ However, all included studies were conducted in populations with overweight or obesity, and there is currently no direct evidence on their effects in individuals with binge eating in the absence of obesity. Identifying mechanisms that are dissociable from weight loss could inform the development of novel pharmacological agents that reproduce the beneficial psychological effects of GLP-1RAs without affecting body weight, allowing their use across the weight spectrum, including in individuals for whom weight loss is neither indicated nor desirable.

Our findings suggest that GLP-1RAs may be a promising *adjunctive* pharmacotherapy for individuals living with binge eating symptoms and obesity. However, as emphasised by our lived experience panel, these drugs should not be viewed as standalone solutions but as one component of a broader, integrated approach: *“It's just a tool—it's not a fix. It will help for the short term, but the psychological* *support needs to be there too.”* While pharmacological treatments may help regulate appetite and reduce loss of control eating, sustainable recovery is likely to depend on concurrent psychological therapies, social support, and policies addressing wider determinants of disordered eating, including societal norms and structural weight bias, as reflected by participants: *“Multiple things got us here, and multiple things will be required to get us out.”* GLP-1RAs may therefore form part of a multidisciplinary strategy integrating medical, behavioural, psychological, and community-level approaches to reduce drivers of binge eating and associated psychological distress.

These findings are particularly relevant in the context of limited global guidance on pharmacological treatments for binge eating and ongoing uncertainty regarding the effectiveness of GLP-1RAs among individuals living with obesity who experience binge eating.

## Contributors

IC: conceptualisation, study design, funding acquisition, literature search, investigation, formal analysis, data curation, software, writing—original draft, review, editing, supervision, and visualisation. IE: study design, investigation, literature search, data curation, methodology, validation, visualisation, writing—original draft. SK: investigation, literature search, writing, and editing. KS: writing and editing; ACW: writing and editing; LW: writing and editing; BS: writing and editing; FS: funding acquisition, investigation, literature search, validation, supervision, writing, review, and editing. CL: supervision, writing, and editing. JM: supervision, writing, and editing. CMB: supervision, writing, and editing. All authors had access to the data and made the final decision to submit the manuscript for publication. IC, IE and FS verified the underlying data.

## Data sharing statement

As this was a meta-analysis of already published randomised controlled trials, no new datasets were produced.

## Declaration of interests

JM has received institutional funding to undertake research trials funded by NovoNordisk, which are unrelated to this work. CM Bulik reports: Pearson Education Inc. (author, royalty recipient) and Orbimed (consultant). SK currently works on a trial funded by the Novo Nordisk Foundation (NNF22SA0080921). The views expressed are those of the author(s) and not necessarily those of the Novo Nordisk Foundation.
