# Supplementary material for: The effectiveness of glucagon-like peptide-1 receptor agonists (GLP-1RAs) on binge eating in patients with obesity: a systematic review and meta-analysis
Source: eClinicalMedicine. 2026 Jul 15;98:104007. doi: 10.1016/j.eclinm.2026.104007 (PMC13523834; doi:10.1016/j.eclinm.2026.104007)
Supplement: Supplementary file 1 [file mmc1.docx]

**The Effectiveness of Glucagon-Like Peptide-1 Receptor Agonists (GLP-1RAs) on Binge Eating in Patients with Obesity: A Systematic Review and Meta-Analysis**

Isobel Emptage^1^, MSc; Stella Kozmér^2^, PhD; Antemeka Cobham-Wilson^1^, M.A; Bernardo Sousa^1^, BBA; Kirsty Sugden^1^, GCSE; Lauren Wheeler^1^, BA(Hons).; Prof Clare Llewellyn^3^, PhD; Janine Makaronidis^4,5,6,7^, PhD; Prof Cynthia Bulik^8,9,10^, PhD; Prof Francesca Solmi^1^, PhD; Ilaria Costantini^1^, PhD

^1^Division of Psychiatry, University College London, London, United Kingdom

^2^Department of Health and Community Sciences, Medical School, University of Exeter, Exeter, United Kingdom

^3^Department of Behavioural Science and Health, University College London, London, United Kingdom

^4^Centre for Obesity Research, Division of Medicine, University College London, London, United Kingdom

^5^NIHR University College London Hospital Biomedical Research Centre, London, UK

^6^Department of Diabetes and Metabolism, Barts Health NHS Trust, London, UK

^7^Blizard Institute, Queen Mary University of London, UK

^8^Department of Medical Epidemiology and Biostatistics, Karolinska Institute, Stockholm, Sweden

^9^Department of Psychiatry, University of North Carolina at Chapel Hill, Chapel Hill, USA

^10^Department of Nutrition, University of North Carolina at Chapel Hill, Chapel Hill, USA

Corresponding author: Ilaria Costantini, [i.costantini@ucl.ac.uk](mailto:i.costantini@ucl.ac.uk), Division of Psychiatry, UCL.

Contents

[**Supplementary Information**	4](#_Toc227683853)

[**PRISMA reporting**	4](#_Toc227683854)

[**Search strategy**	4](#_Toc227683855)

[**Differences from protocol**	4](#_Toc227683856)

[**Supplementary Methods**	4](#_Toc227683857)

[**Addressing effect size multiplicity**	4](#_Toc227683858)

[**Data extraction and management**	4](#_Toc227683859)

[**Further details on data analysis**	5](#_Toc227683860)

[**Assessment of heterogeneity**	6](#_Toc227683861)

[**Post hoc analyses**	6](#_Toc227683862)

[**1. Exclusion of studies at overall high risk of bias**	6](#_Toc227683863)

[**2. Leave-one-out influence analysis**	6](#_Toc227683864)

[**Supplementary Results**	7](#_Toc227683865)

[**Further details on study characteristics**	7](#_Toc227683866)

[**Risk of bias**	7](#_Toc227683867)

[**Subgroup analyses**	7](#_Toc227683868)

[**Post hoc analyses**	7](#_Toc227683869)

[**1. Exclusion of studies at overall high risk of bias**	7](#_Toc227683870)

[**2. Leave-one-out influence analysis**	8](#_Toc227683871)

[**Patient and Public Involvement (PPI) Consultation Summaries**	9](#_Toc227683872)

[**PPI Event 1: Wednesday 17 September 2025**	9](#_Toc227683873)

[**PPI Event 2: Friday 19 September 2025**	10](#_Toc227683874)

[**Supplementary Tables**	12](#_Toc227683875)

[**Supplementary Figures**	23](#_Toc227683876)

**Supplementary Tables**

[Supplementary Table 1. PRISMA 2020 checklist.	12](#_Toc227683877)

[Supplementary Table 2. Search terms used in this systematic review.	14](#_Toc227683878)

[Supplementary Table 3. Inclusion and exclusion criteria applied when considering studies for our systematic review.	15](#_Toc227683879)

[Supplementary Table 4. Working definitions of primary and secondary outcomes.	16](#_Toc227683880)

[Supplementary Table 5. Formulas used to compute the effect estimates included in the meta-analysis.	17](#_Toc227683881)

[Supplementary Table 6. Characteristics of ongoing trials.	18](#_Toc227683882)

[Supplementary Table 7. A table to show GLP-1 receptor agonist weight-loss drugs.	19](#_Toc227683883)

[Supplementary Table 8. Comparator conditions used in our included studies.	20](#_Toc227683884)

[Supplementary Table 9. A table to show assessors’ judgement for the RoB 2.0 risk of bias for each domain and overall, for the primary outcome of each included study.	22](#_Toc227683885)

**Supplementary Figures**

[Supplementary Figure 1. Decision tree indicating strategies to address multiplicity in effect sizes induced by multiple sources.	23](#_Toc227683893)

[Supplementary Figure 2. Meta-regressions for continuous covariates on combined binge eating-related outcomes.	24](#_Toc227683894)

[Supplementary Figure 3. Subgroup analysis of baseline binge eating disorder on primary binge eating-related outcomes.	26](#_Toc227683895)

[Supplementary Figure 4. Subgroup analysis of baseline diabetes on primary binge eating-related outcomes.	28](#_Toc227683896)

[Supplementary Figure 5. Sensitivity analysis excluding studies at overall high risk of bias for the combined primary outcome	29](#_Toc227683897)

[Supplementary Figure 6. Leave-one-out sensitivity analysis for the combined primary outcome	30](#_Toc227683898)

# **Supplementary Information**

## **PRISMA reporting**

We followed the Preferred Reporting Items for Systematic Reviews and Meta-Analyses (PRISMA) 2020 guidelines. A completed PRISMA checklist can be found in **Supplementary Table 1**.

## **Search strategy**

Keywords were combined using Boolean operators and four MeSH terms were used: “Glucagon-Like Peptide-1 Receptor Agonists”, “Glucagon-Like Peptide-1", “Glucagon-Like Peptide-1 Receptor” and “Randomized Controlled Trials”. Remaining terms were searched using text-word fields and these search terms can be seen in **Supplementary Table 2**. Search terms were tailored for each database to account for differences in indexing and syntax; for example database-specific subject headings were used where available and free-text terms were adjusted to match platform requirements such as phrase searching and truncation.

## **Differences from protocol**

While performing title and abstract screening, we expanded our participant level exclusion criteria to exclude studies of participants with genetic disorders in addition to chronic conditions that are not cardiovascular or metabolic, such as Alzheimer’s and Prada Willi Syndrome. This was to maximise comparability of participants across studies for meta-analysis.

We also said that we would have performed a network meta-analysis, however, due to the number of studies included this was not possible and a traditional random-effect meta-analysis was conducted instead. We also prespecified the use of funnel plots to assess publication bias. However, because relatively few studies were available and all outcomes were continuous, we did not formally test for small‐study effects. In this context, Egger’s test is known to be unreliable for continuous outcomes and can yield misleading results.(1)

# **Supplementary Methods**

## **Addressing effect size multiplicity**

Effect size multiplicity was addressed using both convergent and divergent strategies, depending on the source of multiplicity, the available data, and the aims of the research question (**Supplementary Figure 1**).

For each study and outcome, we applied a prespecified hierarchy to derive effect sizes. We first used author-reported mean-change differences when these were available with an associated standard error (SE) or 95% confidence interval (CI). If mean-change differences were not reported, we derived change scores from baseline and follow-up arm-level means and standard deviations (SDs). When studies reported multiple follow-up assessments, we selected the earliest assessment after the intervention. Where multiple scales were used for the same construct within a study, we preferentially selected the instrument most frequently used for that construct across studies.

When studies reported more than one type of outcome estimate (e.g., raw means/SDs, mean changes, or regression-based treatment effects), we extracted the most statistically robust information following a predefined hierarchy. When both baseline and post-intervention arm-level means and SDs were available, we calculated arm-specific mean changes and used these to compute a between-arm difference in mean change, subsequently standardised to Hedges’ g. When studies reported arm-level mean changes and SEs directly, we used these preferentially. Studies reporting only post-intervention raw mean differences, with an associated SE or 95% CI, were included only in the loss-of-control eating raw mean-difference analysis and not in the standardised Hedges’ g analyses. This reflected the heterogeneity of reporting for CoEQ item 19 and the decision not to standardise post-intervention mean-difference-only results for the combined primary outcome.

Across all scenarios, standardised effect sizes (when used) were converted to Hedges’ g, and sampling variances were computed using established metafor-compatible formulas.

When combining the three primary outcomes into a single meta-analysis, some studies contributed more than one binge-eating–related measure. To avoid double counting, we applied a prespecified hierarchy: binge eating was prioritised, followed by loss-of-control eating, and then eating disinhibition. For each study, only the highest-priority measure was retained in the combined model.

## **Data extraction and management**

Raw mean changes were calculated when studies provided baseline and follow-up means. SDs of change were taken directly from study reports when available, derived from reported SEs where possible, or, when unavailable, imputed using a prespecified correlation coefficient of r = 0.5 between baseline and follow-up in line with established guidance(2). To assess the robustness of the pooled estimate to this assumption, a sensitivity analysis was conducted by varying r across three plausible values: 0.25 (low correlation), 0.50 (primary analysis value), and 0.75 (high correlation). All other analytic parameters were held constant across iterations. Studies that directly reported change-score standard errors or between-group mean differences with confidence intervals were unaffected by this assumption and contributed identical estimates across all iterations. When means and SDs were unavailable but medians and interquartile ranges were reported, we approximated means and SDs using established conversion formulas.

For loss-of-control eating outcomes reported as post-intervention mean differences with 95% confidence intervals, we derived the SE from the confidence limits using:

$$SE=\frac{Upper-Lower}{2*1.96}$$

Variance was calculated as *SE^2^*.

Reported and derived change scores were combined into a harmonised dataset. Duplicate records were resolved by retaining the observation with the most complete and precise SE information. Any remaining missing SEs were imputed using the mean SE for that construct and arm across the dataset.

All variables required for the meta-analytic models, including mean change, SE, variance, follow-up classification, intervention characteristics, and study identifiers, were harmonised into a single analysis-ready dataset (see **Supplementary Table 3** for formulas used).

## **Further details on data analysis**

For each study contributing to the standardised meta-analysis, we derived an intervention–control effect size based on the difference in mean change from baseline to follow-up. We prioritised author-reported change scores where available; otherwise, change scores were calculated from reported baseline and post-intervention means. Missing SDs of change were handled as described above. For each comparison, we calculated the pooled SD of change across intervention and control arms using the standard two-group pooled variance formula.

Standardised mean differences were first calculated as Cohen’s d, defined as the difference in mean change between intervention and control groups divided by the pooled SD, and then converted to Hedges’ g using the standard small-sample correction. Sampling variances were calculated using the conventional formula incorporating group sample sizes and the squared effect size. These Hedges’ g estimates and their variances were used in all standardised meta-analyses. Random-effects models were fitted using restricted maximum likelihood (REML), and we report pooled Hedges’ g with 95% confidence intervals (CIs), between-study variance (τ²), the proportion of total variability attributable to heterogeneity (I²), and Cochran’s Q statistic with p-value.

For the combined primary outcome, which synthesised binge eating, loss of control eating, and eating disinhibition within an overall analysis, only studies reporting change-from-baseline values for CoEQ item 19 were eligible, ensuring that all constructs entering the combined model were standardised in an equivalent manner. Only standardised change-score effect sizes contributed to this analysis.

In contrast, the loss-of-control-eating-specific meta-analysis was designed to include the full range of eligible studies, including those reporting only post-intervention means or post-intervention mean differences. Loss of control eating was assessed using item 19 of the Control of Eating Questionnaire (CoEQ), which measured the same construct across trials but was reported on different response scales. Most studies used a 100-mm visual analogue scale (VAS), whereas one trial (3) used a 0–10 numerical rating scale. To ensure comparability, values from Wharton et al.(3) were multiplied by 10, converting the 0–10 scale to the 0–100 VAS metric used in the other trials. This rescaling was applied to means, SDs, and confidence limits for estimated treatment differences. Because the outcome was reported as a mixture of post-intervention scores and change-from-baseline estimates, results for this analysis were synthesised as raw mean differences (intervention minus control), preserving interpretability on the original scale.(3)

We did not assess publication bias using funnel plots or trim-and-fill methods, as the limited number of included studies would make these approaches underpowered, and the presence of substantial heterogeneity and intervention-related differences would further complicate their interpretation.(4)

## **Assessment of heterogeneity**

Heterogeneity was assessed with 𝜏^2^, quantifying the variance of the true effect sizes, and the *I^2^* statistic, representing the percentage of variability in the effect sizes not accounted for by sampling error. Heterogeneity was interpreted as low (25-50%), medium (50-75%) or high (>75% *I^2^*).

## **Post hoc analyses**

To assess the robustness of the pooled estimate for the combined primary outcome, we conducted two post hoc sensitivity analyses that had been recommended at review stage.

### **1. Exclusion of studies at overall high risk of bias**

A second sensitivity analysis excluded studies judged to be at overall high risk of bias based on the ROB-2 tool. Risk-of-bias ratings were derived from the prespecified ROB-2 assessments. The combined primary outcome meta-analysis was then repeated using the same random-effects REML model as the primary analysis.

We compared the pooled effect size, confidence interval, and heterogeneity estimates with those from the main analysis.

### **2. Leave-one-out influence analysis**

We conducted a leave-one-out analysis using the leave1out() function from the metafor package in R. This procedure re-estimates the random-effects meta-analysis model iteratively after removing one study at a time, allowing evaluation of whether any single study disproportionately influenced the pooled effect estimate, confidence interval, or heterogeneity statistics.

For each iteration, we extracted the pooled Hedges’ g, standard error, 95% confidence interval, Cochran’s Q statistic, tau², and I². Results were tabulated and graphically displayed, with the pooled estimate from the full model shown as a reference line.

### **3. Assumed Correlation for SD Change Imputation**

As detailed above in the ‘data extraction and management section’, we conducted a post-hoc sensitivity analysis to assess the robustness of the pooled estimate to the assumption that the correlation between baseline and follow-up measurements was 0.50. We thus re-ran the analyses varying r across three plausible values: 0.25 (low correlation), 0.50 (primary analysis value), and 0.75 (high correlation).

# **Supplementary Results**

## **Further details on study characteristics**

Binge eating and related symptoms were assessed using validated self-report or clinician-rated instruments, including the Binge Eating Scale (BES)(5), Eating Disorder Examination Questionnaire (EDE-Q)(6), 18-item or the revised 18-item Three-Factor Eating Questionnaire (TFEQ)(7–9), Eating Inventory (EI)(10), and loss of control eating was assessed using only item 19 “Generally, how difficult has it been to control your eating?” from the Control of Eating Questionnaire (CoEQ)(8) (see **Supplementary Table 8** for details on subscales or items used). We included nine studies that reported sufficient information to be included in the meta-analysis reporting on binge eating, of which two(11,12) reported on binge eating using the EDE-Q, and seven (13–19) using the BES; 13 studies were included having reported on eating disinhibition or uncontrolled eating, three(11,20) of which using the Eating Inventory, two(16,21–23) the TFEQ, and two (24,25) the TFEQ-R18V2; five studies(3,26–29) included reporting loss of control eating using item 19 from the COEQ.

For secondary outcomes, 13 studies reported sufficient data on cognitive or dietary restraint to be included in the meta-analysis. Cognitive restraint was measured using the EI,(11,12,20,30,31) and different versions of the TFEQ.(16,21–25,32,33) Four studies reported sufficient details on emotional eating for the meta-analysis, all measured emotional eating with different versions of the TFEQ.(14,22–25) Only one study assessed weight and shape concerns; therefore, this outcome was not meta-analysed.(12) One study reported measuring relevant outcome measures (i.e., eating disinhibition, cognitive restraint, emotional eating) but did not provide sufficient information to be included in the meta-analysis.(34)

## **Risk of bias**

All of our included studies were rated either high risk of bias or some concerns (**Supplementary Table 9**). This is largely due to the side effect profile of GLP-1RAs in relation to the measurement of the outcome. In some studies, gastrointestinal side effects were reported more frequently in the intervention group, and even in studies that do not explicitly report this, functional unblinding of participants was likely even in double-blind trials. This is due to the likelihood that participants could infer treatment allocation based on the drastic effect on satiety and the gastrointestinal side effects. Pertinently, most of the comparators used in our included studies were placebo rather than another active weight loss drug, increasing the chances of functional unblinding. To add to this, the measures often used to assess binge eating are self-reported and expectation-sensitive, so knowledge of intervention allocation could alter self-reported sensations and eating behaviours.

Additionally, missingness in outcome data was almost certainly missing not at random due to gastrointestinal side effects being reported as a reason for participant drop out in some studies and plausibly these side effects may be the reason for drop out in others. The side effects of GLP-1RAs such as nausea may correlate with disordered eating patterns such as reducing food intake. Missingness is also plausibly related to treatment failure, as individuals with less appetite suppression may be more likely to discontinue. Another reason that the risk of bias was high or of some concerns in all studies is that while some studies described their analysis as following intention-to-treat or a modified intention-to-treat, they excluded participants who had not adhered to at least one dosage. This could have biased the analysis. Finally, most of the studies reported binge eating symptoms only as a secondary or exploratory outcome and in some instances, these had not been pre-registered or had been pre-registered but not reported. Possibly due to these reasons, the reporting was poor at times with no numerical values being reported even after authors were contacted.

## **Subgroup analyses**

We did not find strong evidence that any of the continuous study- or participant-level characteristics—including mean age (k=20, β=-0·004 [95%CI –0·04 to 0·03], p=0·83), percentage of female participants (k=20, β=0·004 [–0·01 to 0·02], p=0·57) or of White participants (k=15, β= –0·007 [–0·03 to 0·02], p=0·54), risk-of-bias scores (k=21, β= –0·05 [–0·12 to 0·03], p=0·23), baseline BMI (k=21, β= –0·06 [–0·15 to 0·04], p=0·20), waist circumference (k=17, β= –0·02 [–0·06 to 0·03], p=0·46), treatment dose (k=19, β=0·07 [–0·14 to 0·29], p=0·51), total duration (k=21, β=0·001 [–0·01 to 0·01], p=0·82) or duration at full dose (k=21, β=0·001 [–0·01 to 0·01], p=0·80),—moderated the intervention effects (**Supplementary Figure 2**).

## **Post hoc / sensitivity analyses**

To assess the robustness of the combined primary outcome findings, we conducted two post hoc sensitivity analyses as recommended by peer reviewers.

### **1. Exclusion of studies at overall high risk of bias**

When studies judged to be at overall high risk of bias were excluded, the pooled effect estimate was slightly attenuated but remained statistically significant and continued to favour intervention (g = –0.38, 95% CI –0.59 to –0.18; k = 10, n = 1,212), compared with the main analysis (g = –0.50, 95% CI –0.72 to –0.28; k = 21, n = 3,048; **Supplementary Figure 5**). Heterogeneity was also reduced (I² = 62.2% vs 86.3%), suggesting that these studies contributed to between-study variability.

### **2. Leave-one-out influence analysis**

Leave-one-out analyses for the combined primary outcome showed that omission of any single study did not materially alter the overall conclusions. Across iterations, pooled effect estimates remained close to the main analysis estimate (g = -0.50) and consistently favoured intervention over control. In all cases, the direction of effect was unchanged, indicating that no individual study disproportionately drove the primary findings (**Supplementary Figure 6**).

The pooled effect estimate was robust across the range of assumed correlations tested. When r = 0.25, the pooled Hedges' g was −0.472 (95% CI: −0.692 to −0.252); at r = 0.50 (primary analysis), g = −0.497 (95% CI: −0.717 to −0.276); and at r = 0.75, g = −0.552 (95% CI: −0.780 to −0.324). The number of studies (k = 21) and total participants (N = 3,048) were identical across all three iterations, confirming that the varying assumption affected only the precision of imputed change-score SDs rather than study inclusion. Heterogeneity estimates were also stable (I² range: 86.3–87.2%; τ² range: 0.204–0.222), and all pooled estimates remained statistically significant (all p < .001). These findings indicate that the primary results are not materially sensitive to the assumed baseline-to-follow-up correlation used in SD imputation.

### **3. Assumed Correlation for SD Change Imputation**

The pooled effect estimate was robust across the range of assumed correlations tested. When r = 0.25, the pooled Hedges' g was −0.47 (95% CI: −0.69 to −0.25); at r = 0.50 (primary analysis), g = −0.50 (95% CI: −0.72 to −0.28); and at r = 0.75, g = −0.55 (95% CI: −0.78 to −0.32). Heterogeneity estimates were also stable (I² range: 86.3–87.2%; τ² range: 0.204–0.222). These findings indicate that the primary results are not materially sensitive to the assumed baseline-to-follow-up correlation used in SD imputation (see **Supplementary Figure 7**).

# **Patient and Public Involvement (PPI) Consultation Summaries**

IC held two online PPI meetings in September 2025 to gather views on the acceptability and perceived concerns surrounding the use of GLP-1RAsfor managing binge eating among individuals living with higher weight. The feedback from these sessions also informed interpretation and dissemination of findings from this systematic review and meta-analysis.

Each session followed a similar format and lasted approximately 90 minutes. IC opened both meetings with a brief introduction outlining the study aims, the purpose of the PPI consultation, and ground rules to support respectful and confidential discussion. She used short ice-breaker activities at the start of each session to encourage participation and foster a supportive, non-judgemental environment. IC then delivered a short presentation summarising the study background, the rationale for examining GLP-1RAs in relation to binge eating, and the key findings of the meta-analysis.

Participants subsequently engaged in facilitated open discussion, sharing reflections on the findings, their acceptability, and their perceived implications for people with both binge eating and higher weight. Discussion focused on potential benefits, concerns, barriers to access, and the importance of combining pharmacological treatment with psychological and social support. To structure feedback and encourage contributions from all participants, IC used interactive Mentimeter questions, including multiple-choice items, ranking exercises, and open-text responses.

IC closed each session by summarising key points raised, inviting final reflections, and explaining how the feedback would inform interpretation of findings, future research priorities, and dissemination activities.

## **PPI Event 1: Wednesday 17 September 2025**

This PPI consultation involved individuals with lived experience of binge eating, disordered eating, and higher weight, focusing on experiences with weight-loss treatments, including GLP-1RAs, and priorities for research and clinical care.

**Lived experiences and early reflections**

Participants described diverse experiences of disordered eating across the life course. Eating behaviours were commonly described as closely linked to mood, stress, relationships, and wider circumstances. There was agreement that binge eating is “*not about the body; it’s about the brain*.”

Participants described constant “*food noise*,” eating in response to a wide range of emotions, and secrecy around eating behaviours. One participant described such shame that they felt they were “*hiding from yourself*.”

There was consensus that binge eating cannot be reduced to hunger or willpower and must be understood holistically.

**Experiences with treatment and support**

Many participants described limited access to psychologically informed treatment for binge eating. Several reported long histories of trying multiple interventions with little sustained benefit and feelings of isolation within healthcare systems.

There was strong agreement that pharmacological or surgical interventions should be combined with psychological support. One participant stated that people “*need to adjust their thinking – it’s the brain that needs focus.*”

Participants with experience of GLP-1RAs described benefits including reduced compulsive eating and quieter food-related thoughts. Some used strong language, such as “*it turned off something in my brain*” and “*it saved my life*.” At the same time, concerns about affordability and sustainability were common.

**Barriers to access and eligibility**

Cost and reliance on private prescriptions were again highlighted as major barriers. Participants also described exclusion based on eligibility criteria and frustration with limited understanding among healthcare professionals of both binge eating and overweight/obesity.

Long waiting times, medication shortages, and inequalities in access were perceived as significant obstacles to care.

**Psychological and societal aspects**

Participants emphasised the psychological and societal drivers of binge eating, including environmental food cues and social contexts. One participant noted that binge eating “*has to do with the supermarkets around you, with your emotions, with society, with all of that.”*

Dietary restriction was frequently described as worsening binge eating through rebound effects. There was agreement that approaches focused solely on diet or weight are insufficient.

**Acceptability, hopes, and concerns regarding GLP-1RAs**

Most participants viewed GLP-1RAs as acceptable, particularly where they reduced loss of control and food preoccupation. Hopes included improved mental wellbeing, reduced distress, and better quality of life.

Concerns included cost, side-effects, uncertainty about long-term safety, and fear of weight- and binge eating symptoms- rebound after stopping medication. One participant noted that “*without adequate support, all of a sudden, you could go back to where you were two years ago.*”

Participants emphasised that acceptability depends on GLP-1RAs being offered within holistic care pathways. One participant summarised this clearly: *“It’s just a tool – it’s not a fix. It will help for the short term, but the psychological support needs to be there too.”*

**Research priorities and advice for researchers**

Participants highlighted the need for research addressing biological, psychological, and societal contributors to binge eating, including appetite regulation, brain mechanisms, emotional coping, and social connection. Some expressed interest in treatments that reduce binge eating without focusing on weight loss.

Participants encouraged researchers to prioritise outcomes beyond weight change and to recognise individual differences in treatment response.

**Closing reflections**

Participants valued the opportunity to contribute to research and felt that lived-experience engagement can reduce isolation and improve understanding. Many described the session as empowering and appreciated being involved in shaping future research priorities.

## **PPI Event 2: Friday 19 September 2025**

This PPI consultation brought together individuals with lived experience of binge eating, disordered eating, and higher weight to discuss experiences with weight-management treatments, including GLP-1RAs, and to reflect on the acceptability and perceived risks of using these medications for binge eating.

**Lived experiences and early reflections**

Participants described varied and often long-standing difficulties with binge eating and weight management. Some reported that weight-related difficulties were recognised before binge eating was identified as a central issue, while others described binge eating as the primary difficulty, later associated with weight gain. Several participants noted that binge eating was not always formally diagnosed, despite persistent symptoms, and that not all individuals identified with the label of an eating disorder.

There was a shared perception that binge eating disorder receives less attention, funding, and prioritisation than other eating disorders. One participant reflected that binge eating “*often feels overlooked compared to other eating disorders*,” contributing to delayed recognition and support.

Participants emphasised that binge eating cannot be understood in isolation. There was agreement that eating behaviours are shaped by psychological, social, and developmental factors, and that “*it’s never just about food*.”

**Experiences with treatment and support**

Participants reported a wide range of treatment experiences, including GLP-1RAs, bariatric surgery, and other medical treatments. Some described rapid weight regain after discontinuing GLP-1RAs, which they found discouraging and distressing. Others described fragmented care, where medical and psychological treatments were not well integrated.

Several participants highlighted the profound impact of binge eating and higher weight on daily functioning and quality of life. One participant noted that binge eating can affect “*every part of your life, not just your health*.”

There was a shared view that treatment is often offered too late and rarely from a preventative perspective. Earlier access to integrated care was viewed as potentially beneficial.

Participants also highlighted the lack of clear pathways to manage binge eating while waiting for psychological treatment. In this context, medication was sometimes viewed as a way to stabilise symptoms and reduce distress during long waiting periods.

**Barriers to access and eligibility**

Cost was consistently identified as a major barrier, particularly given the need for long-term treatment and reliance on private prescriptions. Participants expressed concern about sustainability of access over time.

Eligibility criteria were also described as restrictive. Some participants reported being excluded due to past treatments or eating-related histories. One participant commented that answering honestly to screening questions could lead to exclusion.

Participants also described healthcare encounters that felt overly simplistic or focused on willpower. Advice such as “*just eat less*” was seen as failing to reflect the complexity of binge eating and higher weight, and as increasing shame and guilt.

**Psychological, biological, and societal aspects**

Participants expressed strong interest in understanding the biological mechanisms underlying binge eating, including appetite regulation and gut–brain signalling. One participant said they wanted to know whether they were “*biologically predisposed to this*.”

Understanding biology was viewed as important in reducing shame. One participant reflected that learning about appetite regulation “*takes the guilt away and moves it away from willpower*.”

Participants also highlighted societal stigma around body size and eating behaviours. Shame and judgement were described as barriers to help-seeking. One participant summarised this as “*a complete lack of understanding of what people actually go through*.”

**Acceptability, hopes, and concerns regarding GLP-1RAs**

GLP-1RAs were generally viewed as acceptable and, for some, the most effective treatment currently available. Benefits were often described in psychological terms. Participants reported reduced food preoccupation, with one noting that the medication “*switches something off – the food stops being at the forefront of your mind*,” and another asking, *“Is this what normal people feel like?”*

Some participants also described perceived improvements in mood and health. Others felt the medication helped prevent reaching “*the point where the body can’t cope anymore*.”

Concerns were raised about side-effects, including severe gastrointestinal symptoms that affected daily functioning. One participant described this as “*completely limiting your life*.” Participants also raised concerns about long-term safety and uncertainty about use in different life circumstances.

There was concern that psychological support might be reduced if GLP-1RAs are treated as first-line solutions. Participants consistently emphasised that medication should be viewed as a tool rather than a cure. One participant stated: *“Multiple things got us here, and multiple things will be required to get us out.”*

Differences in individual responses to medication were also discussed, reinforcing the need for personalised approaches and better understanding of underlying mechanisms.

**Priorities for future research**

Participants expressed concern about limited research investment in binge eating disorder, which one participant described as “*very unfair*.” There was strong support for research adopting a holistic approach that integrates biological, psychological, and environmental factors.

Specific interests included appetite-regulating hormones, daily patterns of hunger and satiety, and environmental influences on eating behaviour. Several participants expressed interest in contributing to future research, including clinical trials.

**Closing reflections**

Participants valued having a space to share experiences that are often overlooked or misunderstood. Many described the discussion as validating and appreciated the opportunity to contribute to research aimed at improving understanding and care.

# **Supplementary Tables**

Supplementary Table 1. PRISMA 2020 checklist.

| Section and Topic | Item # | Checklist item | Location  where item is reported |
| --- | --- | --- | --- |
| TITLE | | |  |
| Title | 1 | Identify the report as a systematic review. | Page 1, Title page |
| ABSTRACT | | |  |
| Abstract | 2 | See the PRISMA 2020 for Abstracts checklist. | Page 2, Abstract |
| INTRODUCTION | | |  |
| Rationale | 3 | Describe the rationale for the review in the context of existing knowledge. | Page 4, Introduction |
| Objectives | 4 | Provide an explicit statement of the objective(s) or question(s) the review addresses. | Page 7, Introduction |
| METHODS | | |  |
| Eligibility criteria | 5 | Specify the inclusion and exclusion criteria for the review and how studies were grouped for the syntheses. | Page 8 and Supplementary Table 3  Methods – Eligibility Criteria |
| Information sources | 6 | Specify all databases, registers, websites, organisations, reference lists and other sources searched or consulted to identify studies. Specify the date when each source was last searched or consulted. | Page 8  Methods – Search strategy |
| Search strategy | 7 | Present the full search strategies for all databases, registers and websites, including any filters and limits used. | Appendix page 4 and Supplementary Table 2 |
| Selection process | 8 | Specify the methods used to decide whether a study met the inclusion criteria of the review, including how many reviewers screened each record and each report retrieved, whether they worked independently, and if applicable, details of automation tools used in the process. | Page 8  Methods – Study selection |
| Data collection process | 9 | Specify the methods used to collect data from reports, including how many reviewers collected data from each report, whether they worked independently, any processes for obtaining or confirming data from study investigators, and if applicable, details of automation tools used in the process. | Page 9  Methods – Data extraction |
| Data items | 10a | List and define all outcomes for which data were sought. Specify whether all results that were compatible with each outcome domain in each study were sought (e.g. for all measures, time points, analyses), and if not, the methods used to decide which results to collect. | Page 9, Supplementary Table 4,  Methods – Outcomes |
|  | 10b | List and define all other variables for which data were sought (e.g. participant and intervention characteristics, funding sources). Describe any assumptions made about any missing or unclear information. | Table 1  Methods – Data extraction |
| Study risk of bias assessment | 11 | Specify the methods used to assess risk of bias in the included studies, including details of the tool(s) used, how many reviewers assessed each study and whether they worked independently, and if applicable, details of automation tools used in the process. | Page 9  Methods – Risk of bias |
| Effect measures | 12 | Specify for each outcome the effect measure(s) (e.g. risk ratio, mean difference) used in the synthesis or presentation of results. | Methods – Statistical analysis and Appendix page 5 |
| Synthesis methods | 13a | Describe the processes used to decide which studies were eligible for each synthesis (e.g. tabulating the study intervention characteristics and comparing against the planned groups for each synthesis (item #5)). | Methods – Statistical analysis and Appendix page 5 |
|  | 13b | Describe any methods required to prepare the data for presentation or synthesis, such as handling of missing summary statistics, or data conversions. | Methods – Statistical analysis and Appendix page 5 |
|  | 13c | Describe any methods used to tabulate or visually display results of individual studies and syntheses. | Methods – Statistical analysis and Table 1 |
|  | 13d | Describe any methods used to synthesize results and provide a rationale for the choice(s). If meta-analysis was performed, describe the model(s), method(s) to identify the presence and extent of statistical heterogeneity, and software package(s) used. | Methods – Statistical analysis Page 10 |
|  | 13e | Describe any methods used to explore possible causes of heterogeneity among study results (e.g. subgroup analysis, meta-regression). | Methods – Statistical analysis Page 10 |
|  | 13f | Describe any sensitivity analyses conducted to assess robustness of the synthesized results. | Methods – Statistical analysis Page 11 |
| Reporting bias assessment | 14 | Describe any methods used to assess risk of bias due to missing results in a synthesis (arising from reporting biases). | Methods – Statistical analysis Page 11, and Appendix Page 7 |
| Certainty assessment | 15 | Describe any methods used to assess certainty (or confidence) in the body of evidence for an outcome. | Page 11 |
| RESULTS | | |  |
| Study selection | 16a | Describe the results of the search and selection process, from the number of records identified in the search to the number of studies included in the review, ideally using a flow diagram. | Page 12 |
|  | 16b | Cite studies that might appear to meet the inclusion criteria, but which were excluded, and explain why they were excluded. | NA |
| Study characteristics | 17 | Cite each included study and present its characteristics. | Table 1 and Results Section lines 241-271 |
| Risk of bias in studies | 18 | Present assessments of risk of bias for each included study. | Figure 3 and Supplementary Table 9 |
| Results of individual studies | 19 | For all outcomes, present, for each study: (a) summary statistics for each group (where appropriate) and (b) an effect estimate and its precision (e.g. confidence/credible interval), ideally using structured tables or plots. | Figures 4 and 5 |
| Results of syntheses | 20a | For each synthesis, briefly summarise the characteristics and risk of bias among contributing studies. | Figure 3, main text pp.12-13, Supplementary Material p. 6 and Supplementary Table 9 |
|  | 20b | Present results of all statistical syntheses conducted. If meta-analysis was done, present for each the summary estimate and its precision (e.g. confidence/credible interval) and measures of statistical heterogeneity. If comparing groups, describe the direction of the effect. | Page 14 |
|  | 20c | Present results of all investigations of possible causes of heterogeneity among study results. | Page 14 |
|  | 20d | Present results of all sensitivity analyses conducted to assess the robustness of the synthesized results. | Page 15 and Appendix Page 7 |
| Reporting biases | 21 | Present assessments of risk of bias due to missing results (arising from reporting biases) for each synthesis assessed. | Supplementary Table 9 |
| Certainty of evidence | 22 | Present assessments of certainty (or confidence) in the body of evidence for each outcome assessed. | NA |
| DISCUSSION | | |  |
| Discussion | 23a | Provide a general interpretation of the results in the context of other evidence. | Page 15 |
|  | 23b | Discuss any limitations of the evidence included in the review. | Page 15 |
|  | 23c | Discuss any limitations of the review processes used. | Page 15 |
|  | 23d | Discuss implications of the results for practice, policy, and future research. | Page 18 |
| OTHER INFORMATION | | |  |
| Registration and protocol | 24a | Provide registration information for the review, including register name and registration number, or state that the review was not registered. | Page 2 |
|  | 24b | Indicate where the review protocol can be accessed, or state that a protocol was not prepared. | Page 2 |
|  | 24c | Describe and explain any amendments to information provided at registration or in the protocol. | Supplementary Material p 4 |
| Support | 25 | Describe sources of financial or non-financial support for the review, and the role of the funders or sponsors in the review. | Page 11 |
| Competing interests | 26 | Declare any competing interests of review authors. | Page 20 |
| Availability of data, code and other materials | 27 | Report which of the following are publicly available and where they can be found: template data collection forms; data extracted from included studies; data used for all analyses; analytic code; any other materials used in the review. | NA |

Supplementary Table 2. Search terms used in this systematic review.

| **PICOS** | **Search terms** |
| --- | --- |
| Population: human participants without chronic health conditions that are not cardiovascular or metabolic | NA |
| Intervention: Glucagon-Like Peptide-1 Receptor Agonists | “Glucagon-Like Peptide-1 Receptor Agonists”, “GLP-1 receptor agonist”, “Glucagon-Like Peptide 1”, “GLP 1”, “Glucagon-Like Peptide-1 Receptor”, “ozempic”, “Novo Nordisk”, “Rybelsus”, “Wegovy”, “Semaglutide”, “Exenatide”, “Bydureon”, “Byetta”, “Exendin*”, “Liraglutid*”, “Saxenda”, “Victoza”, “Xultrophy”, “Lixisenatide”, “Dulaglutide”, “Albiglutide”, “Adlyxin”, “Tanzeum”, “Trulicity”, “Tirzepatide”, “Glutazumab” |
| Comparator condition: any control group | NA |
| Outcome: BED or related symptoms of binge eating | NA |
|  |  |
| Study design: Randomised Controlled Trials | “Randomized controlled trials”, “RCT*”, “randomi*ed control* trial”, “controlled clinical study”, “random* allocat*”, “cross-over trial*” |

*Note.* The search was not restricted on population, comparator condition, or outcome to increase the sensitivity of the search, BED = binge eating disorder.

Supplementary Table 3. Inclusion and exclusion criteria applied when considering studies for our systematic review.

| **Domain** | **Eligibility** |
| --- | --- |
| Population | Human participants of any age, gender, or ethnicity were included. Studies selectively sampling patients with chronic health conditions that were not cardiovascular or metabolic were excluded, including those where such patients comprised 25% or more of the total sample. These conditions included, but were not limited to, cancer, HIV, Alzheimer’s and Prada Willi Syndrome. This was done to maximise the exchangeability assumption for the planned meta-analysis, as well as the credibility of the transitivity assumption in the case of a network meta-analysis |
| Intervention | Any weight-loss drug targeting GLP-1 was included. There were no restrictions on dosage, duration, nor mode of delivery. Drugs with dual targets were eligible, including Tirzepatide which targets GIP as well as GLP-1. Infusions of the endogenous hormone GLP-1 were excluded, in order to maintain a consistent and focused scope of our review on weight-loss drugs specifically without introducing heterogeneity in intervention mechanisms |
| Comparator | Given the paucity of research on GLP-1 receptor agonists for our outcome and to enhance the scope of our findings, any control group comparator was eligible. This included TAU which is typically weight-loss management programs and behavioural support, wait list, placebo, no intervention, or any active interventions such as pharmacological, behavioural, dietary, psychological or a combination of these |
| Outcomes | Trials measuring the primary outcome of binge eating disorder or related symptoms of binge eating such as loss-of-control, disinhibited, or uncontrolled eating were included. Eligible measurements included the count of binge- eating episodes, clinical diagnoses or administration of validated questionnaires such as the EDE-Q, BES or TFEQ. Studies that only measured our secondary outcome of emotional eating, restrictive eating behaviours, or body-image concerns were not included |
| Study design | RCTs using both individual (i.e., patients) and group (i.e., hospitals) units of randomisation were included. Cross- over trials also met our inclusion criteria. Animal, qualitative, non-randomised cross-sectional and longitudinal observational studies were excluded. Only trials conducted from 2005 were deemed eligible as this was the year of the first GLP-1 receptor agonist approval and could be conducted in any country or setting including hospitals and community-based care. Studies published in English, in peer-reviewed journal articles or as theses and dissertations, or results posted on trial registry pages without an associated publication were included. Eligible, ongoing trials were saved and tabulated in **Supplementary Table 6**. Systematic reviews and meta-analyses were excluded but hand searched for studies meeting eligibility, and conference proceedings were excluded but authors contacted to request full missing data or associated publications |

***Note.*** GIP = glucose-dependent insulinotropic polypeptide. TAU = treatment as usual. EDE-Q = eating disorder examination questionnaire, BES = binge eating scale, TFEQ = three factor eating questionnaire, RCT = randomised controlled trial.

Supplementary Table 4. Working definitions of primary and secondary outcomes.

| **Construct** | **Definition** | **Scales included in the review** |
| --- | --- | --- |
| **Primary outcomes** |  |  |
| Binge eating | The experience of consuming large amounts of food despite a desire not to. This refers to the overall difficulties in regulating eating.(35) | **Binge Eating Scale (BES):** 16-item self-report assessing behavioural (e.g. rapid or excessive eating) and cognitive–emotional symptoms (e.g. guilt, feeling out of control). Total score 0–46, higher scores indicate more severe binge eating. **Eating Disorder Examination Questionnaire (EDE-Q):** Self-report version of the EDE interview assessing frequency of binge-type episodes. Higher scores reflect greater symptom severity. |
| Uncontrolled and disinhibited eating | The tendency to overeat in response to different stimuli. Opportunistic eating in an obesogenic environment(36). Higher disinhibition reflects higher susceptibility to overeat | **Three-Factor Eating Questionnaire (TFEQ); also known as the Eating Inventory (EI):** A 51-item widely used and validated self-report questionnaire to assess key behavioural and psychological components of eating, with a separate subscale for disinhibited eating.  **Three-Factor Eating Questionnaire [TFEQ-R18 V2]:** A shortened, revised version of the 51-item TFEQ. This version is often used in large clinical trials and retains 3 subscales with ‘disinhibition’ renamed as ‘uncontrolled eating’. |
| Loss-of-control eating | The subjective feeling an individual has that they cannot stop eating or cannot control how much they are eating | **Control of Eating Questionnaire (COEQ):** Self-report questionnaire designed to assess subjective experiences around eating. Specifically, we focused on item 19 ‘Generally, how difficult has it been to control your eating?’. |
| **Secondary Outcomes** |  |  |
| Restrictive eating behaviour | The conscious intent and perceived ability to restrict food intake to influence body weight or shape(37) reflects dieting cognitions and efforts rather than actual restriction. Includes cognitive restraint and dietary restraint which are the cognitive effort and behavioural attempts to restrict food intake.(37) Importantly, cognitive/dietary restraint reflects the intention (cognitive) or effort (behavioural) to limit food intake, whereas eating restriction refers to the actual reduction in energy intake, and these constructs are conceptually distinct and not necessarily strongly correlated.(38–40) | **Eating Disorder Examination Questionnaire (EDE-Q):** Specifically, the dietary restraint subscale of this self-report questionnaire where items are scored on a 7-point Likert scale (0 = no days, 6 = every day). Higher scores indicate greater dietary restraint.  **Three-Factor Eating Questionnaire (TFEQ); also known as the Eating Inventory (EI):** The cognitive restraint subscale of this 51-item questionnaire contains 21 items.  **Three-Factor Eating Questionnaire [TFEQ-R18 V2]:** The cognitive restraint subscale of this 18-item shortened version of the TFEQ contains 6-items and focuses on the core aspects of cognitive restraint. |
| Emotional eating | Eating in response to emotional states—particularly negative emotions such as anxiety, sadness, or stress—rather than physiological hunger.(41) | **Three-Factor Eating Questionnaire [TFEQ-R18 V2]:** This emotional eating subscale only appears in the shortened version of the TFEQ and is made up of 3 items. Scores are summed, with higher scores reflecting greater emotional eating. |
| Weight and shape concerns | This encapsulates weight and shape concerns. Weight concerns are a preoccupation with body weight, desire to lose weight, dissatisfaction with current weight, and distress or avoidance related to weighing. Shape concerns are negative thoughts, feelings, and behaviours related to body shape, including preoccupation with body size, fear of weight gain, body dissatisfaction, and avoidance of body exposure. | **Eating Disorder Examination Questionnaire (EDE-Q):** The weight concern subscale is made up of 5 items. An example item is ‘Over the past 28 days, how concerned have you been about gaining weight?’.  **Eating Disorder Examination Questionnaire (EDE-Q):** The shape concern subscale is made up of 8 items. An example item is ‘Over the past 28 days, has your shape influenced how you feel about yourself?’. |

Supplementary Table 5. Formulas used to compute the effect estimates included in the meta-analysis.

| **Parameter** | **Mathematical formulas** |
| --- | --- |
| 1. Standard deviation (SD) | $SD=SE*\surd(N)$ |
| 2. SD | $SD=\frac{HighIQR-LowIQR}{1.35}$ |
| 3. Standard Error (SE) of Mean Difference | $\mathrm{SE}\Delta=SD\Delta/\sqrt{N}$ |
| 4. SE | $SE=\frac{\text{Upper}-\text{Lower}}{2\times1.96},$ |
| 5. Pooled SD | $SD_{p}=\sqrt{\frac{(n_{c}-1)SD_{c}^{2}+(n_{t}-1)SD_{t}^{2}}{n_{c}+n_{t}-2}}$ |
| 5. Mean change | $\Delta\bar{X}=\bar{X}_{\text{follow-up}}-\bar{X}_{\text{baseline}}$ |
| 6. SD of change | *r=0.5*  $SD_{\Delta}=\sqrt{SD_{post}^{2}+SD_{base}^{2}-2r(SD_{post})(SD_{base})}$ |
| 7. Cohen’s d | $d=\frac{M_{t}-M_{c}}{SD_{p}}$ |
| 6. Small-sample correction | $J=1-\frac{3}{4(n_{c}+n_{t}-2)-1}$ |
| 7. Hedges’ g | $g=J\cdot d$ |
| 8. Variance of MD from CI | $Var \left( MD \right)=SE^{2}$ |
| 9. Variance of raw mean difference | $Var \left( MD \right)=\frac{SD_{t}^{2}}{n_{t}}+\frac{SD_{c}^{2}}{n_{c}}$ |
| 9. Variance of d | $Var(d)=\frac{n_{c}+n_{t}}{n_{c}n_{t}}+\frac{d^{2}}{2(n_{c}+n_{t}-2)}$ |
| 10. Variance of g | $Var(g)=J^{2}\cdot Var(d)$ |

Supplementary Table 6. Characteristics of ongoing trials.

| **Trial reference number** | **Study design** | **Sample** | **Intervention** | **Comparator** | **Measures of primary outcome** | **Measures of secondary**  **outcome** | **Current stage** | **Expected completion** |
| --- | --- | --- | --- | --- | --- | --- | --- | --- |
| ClinicalTrials.gov: NCT05548647 | Double-Blind; RCT | Non-diabetic obese individuals  N = NI | Semaglutide plus lifestyle modification for 72 weeks | Placebo plus lifestyle modification | Self-report EI dietary disinhibition; LOCES loss-of- control eating; CoEQ scores; clinician- rated binge-eating frequency using EDE-Q | Self-report EI cognitive restraint scores | Completed; no results posted | Completed 12/05/2025 |
| ClinicalTrials.gov: NCT04854083 | Double-Blind; RCT | Obese type 2 diabetics  N = 50 | 1.34 mg Semaglutide plus lifestyle treatment for 13 weeks | Placebo plus lifestyle treatment | Self-report binge eating and loss-of-control eating using CoEQ, QEWP and BES scores | NA | Unknown status | 08/2025 |
| ClinicalTrials.gov: NCT06243536 | RCT | Type 2 diabetics  N = 60 | Semaglutide plus standard care for 12 weeks | Standard care | Disordered eating behaviour using self-report EAT-26 scores | NA | Unknown status | 31/12/2024 |
| ClinicalTrials.gov: NCT06431308 | Open-label; RCT | Obesity plus an adiposity-related condition  N = 120 | Wegovy or Mounjaro plus nutritional intervention for 20 weeks | Wegovy or Mounjaro plus standard care | Loss-of-control eating using self-report CoEQ scores | NA | Recruiting | 30/03/2026 |
| ClinicalTrials.gov: NCT06710587 | Open-label; RCT | Obesity  N = 470 | GLP-1RA plus coaching | GLP-1RA | Binge eating using BES and uncontrolled eating using TFEQ scores | Emotional eating and cognitive restraint measured by self-report TFEQ scores | Not yet recruiting | 11/04/2027 |
| ClinicalTrials.gov: NCT06691243 | Double-Blind; RCT | Individuals with co-occurring alcohol use disorder and overweight or obesity  N = 46 | 5 mg Tirzepatide | Placebo | Loss-of-control eating using self-report R-BEDS scores | NA | Not yet recruiting | 15/01/2028 |
| ClinicalTrials.gov: NCT07284979 | Partially-blinded; RCT | Overweight  N= NI | Semaglutide once-weekly | Placebo | Loss-of-control eating using self-report CoEQ scores | NA | Recruiting | 17/04/2028 |
| ClinicalTrials.gov: NCT07284511 | Open-label; RCT | Type 1 diabetics  N = 105 | Tirzepatide once-weekly | Automated insulin delivery system | Self-report TFEQ uncontrolled eating scores | Self-report TFEQ emotional eating scores | Not yet recruiting | 01/2028 |
| ClinicalTrials.gov: NCT06847399 | Double-Blind; RCT | Individuals with obesity and binge eating disorder  N = NI | Tirzepatide | Placebo | Change in number of binge-eating episodes | NA | Recruiting | 01/12/2027 |
| ClinicalTrials.gov: NCT06780449 | RCT | Overweight  N= 400 | 2.4 mg Cagrilinitide in combination with Semaglutide | Placebo | Self-report loss-of-control eating CoEQ scores | NA | Active |  |
| ClinicalTrials.gov: NCT06691243 | Double-Blind; RCT | Individuals with cocaine use disorder  N = 40 | Weekly Semaglutide injections up to 2 mg for 16 weeks | Placebo | Loss-of-control eating using self-report G-FCQ-T scores | Emotional eating using the negative reinforcement subscale of the FCQ-T | Recruiting | 01/03/2027 |

***Note.*** EI = eating inventory (10), LOCES = loss of control over eating scale (42), CoEQ = control of eating questionnaire (8), EDE-Q = eating disorder examination (6), QEWP = questionnaire on eating and weight patterns (43), DEBQ = Dutch eating behaviour questionnaire (44), BES = binge eating scale (45), A-EBQ = Auckland eating behaviour questionnaire (46), EAT-26 = eating attitudes test (47), EBQ = eating beliefs questionnaire (48), TFEQ = three factor eating questionnaire (9), R-BEDS = reward-based eating drive scale. Note that the TFEQ is the same measure as the EI.

Supplementary Table 7. A table to show GLP-1 receptor agonist weight-loss drugs.

| **GLP-1 RA/study** | **Commercial name** | **Half-life** | **Dose** | **Frequency** | **Method** | **Date of approval** | **Indication** |
| --- | --- | --- | --- | --- | --- | --- | --- |
| Exenatide | Byetta | 2.4 hrs |  |  |  | April 2005 | Type 2 diabetes |
| Gatta-Cherifi et al. (2024) |  |  | 10 mcg | Twice daily | Subcutaneous injection |  |  |
| Patino et al. (2025) |  |  | 10 mcg | Twice daily | Subcutaneous injection | |  |
| Liraglutide | Victoza; Saxenda | 13 hrs |  |  |  | January 2010 | Type 2 diabetes; weight management |
| Allison et al. (2022) |  |  | 3 mg | Once daily | Subcutaneous injection |  |  |
| Chao et al. (2019) |  |  | 3 mg | Once daily | Subcutaneous injection |  |  |
| Davies et al. (2015) |  |  | 3 mg | Once daily | Subcutaneous injection |  |  |
| Dubé et al. (2020) |  |  | 1.8 mg | Once weekly | Subcutaneous injection |  |  |
| Gudbergsen et al. (2021) |  |  | 3 mg | Once daily | Subcutaneous injection |  |  |
| Jensen et al. (2022) |  |  | 3 mg | Once daily | Subcutaneous injection |  |  |
| Lau et al. (2021) |  |  | 3 mg | Once daily | Subcutaneous injection |  |  |
| McElroy et al. (2024) |  |  | 3 mg | Once daily | Subcutaneous injection |  |  |
| Pi-Sunyer et al. (2015) |  |  | 3 mg | Once daily | Subcutaneous injection |  |  |
| Robert et al. (2015) |  |  | 1.8 mg | Once daily | Subcutaneous injection |  |  |
| Wadden et al. (2013) |  |  | 3 mg | Once daily | Subcutaneous injection |  |  |
| Dulaglutide | Trulicity | 5 d |  |  |  | September 2014 | Type 2 diabetes |
| Da Porto et al. (2020) |  |  | 1.5 mg | Once weekly | NI |  |  |
| Semaglutide | Ozempic; Rybelsus | 1 wks |  |  |  | December 2017 | Type 2 diabetes; weight management |
| Blundell et al. (2017) |  |  | 1 mg | Once weekly | Subcutaneous injection |  |  |
| Friedrichsen et al. (2020) |  |  | 2.4 mg | Once weekly | Subcutaneous injection |  |  |
| Gabe et al. (2024) |  |  | 50 mg | Once daily | Oral |  |  |
| Gibbons et al. (2020) |  |  | 14 mg | Once daily | Oral |  |  |
| Jensterle et al. (2023) |  |  | 1 mg | Once weekly | Subcutaneous injection |  |  |
| Wharton et al. (2023) |  |  | 2.4 mg | Once weekly | Subcutaneous injection |  |  |
| Tirzepatide | Mounjaro | 5 d |  |  |  | December 2022 | Type 2 diabetes; weight management |
| Martin et al. (2024) |  |  | 10 mg | Once weekly | Subcutaneous injection |  |  |
| Snaith et al. (2025) |  |  | 5 mg | Once weekly | Subcutaneous injection | |  |
| Survodutide | NA^1^ | NA^1^ |  |  |  | Not yet approved^1^ | Weight management |
| Blüher et al. (2024) |  |  | 2.7 mg | Once weekly | Subcutaneous injection |  |  |
| Retatrutide | NA ^1^ | NA ^1^ |  |  |  | Not yet approved ^1^ | Weight management |
| Kanu et al. (2025) [1] |  |  | 12 mg | Once weekly | Subcutaneous injection |  |  |
| Kanu et al. (2025) [2] |  |  | 12 mg | Once weekly | Subcutaneous injection |  |  |

***Note.*** The information listed corresponds to the intervention that was carried forward for the meta-analysis of this review, half-life = the amount of time it takes for half of a drug to be eliminated from the body, mcg = microgram, mg = milligram, GLP-1RA = glucagon-like peptide 1 receptor agonist; hours: hrs; wks: weeks; d: days

1 Currently in phase 3 clinical trials

Supplementary Table 8. Comparator conditions used in our included studies.

| **Comparator condition/study** | **Dose** | **Frequency** | **Delivery method** | **Combined intervention** | **Description** |
| --- | --- | --- | --- | --- | --- |
| Placebo |  |  |  |  | An inactive substance or injection with no pharmacological effect, designed to look identical to the study drug |
| Allison et al. (2022) | 6.0 mg | Once daily | Subcutaneous injection |  |  |
| Blüher et al. (2024) | 2.7 mg | Once weekly | Subcutaneous injection |  |  |
| Blundell et al. (2017) | 1.0 mg | Once weekly | Subcutaneous injection |  |  |
| Davies et al. (2015) | 3.0 mg | Once daily | Subcutaneous injection |  |  |
| Dubé et al. (2020) | 1.8 mg | Once weekly | Subcutaneous injection |  |  |
| Friedrichsen et al. (2020) | 2.4 mg | Once weekly | Subcutaneous injection |  |  |
| Gabe et al. (2024) | 50 mg | Once daily | Oral |  |  |
| Gibbons et al. (2020) | 14 mg | Once daily | Oral |  |  |
| Jensen et al. (2022) | 3 mg | Once daily | Subcutaneous injection |  |  |
| Jensterle et al. (2023) | 1 mg | Once weekly | Subcutaneous injection |  |  |
| Kanu et al. (2025) [1] | 12 mg | Once weekly | Subcutaneous injection |  |  |
| Kanu et al. (2025) [2] | 12 mg | Once weekly | Subcutaneous injection |  |  |
| Lau et al. (2021) | 3 mg | Once daily | Subcutaneous injection |  |  |
| Martin et al. (2024) | 10 mg | Once weekly | Subcutaneous injection |  |  |
| McElroy et al. (2024) | 3 mg | Once daily | Subcutaneous injection |  |  |
| Patino et al. (2025) | 0.01 mg | Twice daily | Subcutaneous injection |  |  |
| Snaith et al. (2025) | 5 mg | Once weekly | Subcutaneous injection |  |  |
| Diet, Exercise, lifestyle intervention or IBT |  |  |  |  | Often a combination of a hypocaloric diet and regular moderate intensity physical activity and sometimes using pedometers and food diaries. IBT is a structured, counseling approach to weight management that supports sustainable lifestyle changes |
| Chao et al. (2019) | 15 minutes | 21 sessions (weekly for 4 visits, then every other week for the next 10 sessions. then every 4 weeks for the remaining 7) | NI |  |  |
| Robert et al. (2015) | NI | NI | NI |  |  |
| Placebo plus diet, exercise, lifestyle intervention or IBT |  |  |  |  |  |
| Gatta-Cherifi et al. (2024) | Placebo: 0.01 mg  Lifestyle intervention: NI | Placebo: twice daily  Lifestyle intervention: once monthly | Placebo: subcutaneous injection  Lifestyle intervention: NI |  |  |
| Gudbergsen et al. (2021) | Placebo: 3 mg  Lifestyle intervention: NI | Placebo: once daily  Lifestyle intervention: once weekly | Placebo: subcutaneous injection  Lifestyle intervention: group sessions |  |  |
| Pi-Sunyer et al. (2015) | Placebo: 3 mg  Lifestyle intervention: NI | Placebo: once daily  Lifestyle intervention: NI | Placebo: subcutaneous injection  Lifestyle intervention: individual or group sessions |  |  |
| Wadden et al. (2013) | Placebo: 3 mg  Lifestyle intervention: NI | Placebo: once daily  Lifestyle intervention: NI | Placebo: subcutaneous injection  Lifestyle intervention: NI |  |  |
| Wharton et al. (2023) | Placebo: 2.4 mg  Lifestyle intervention: NI | Placebo: once weekly  Lifestyle intervention: once monthly | Placebo: subcutaneous injection  Lifestyle intervention: in-clinic visits or phone calls |  |  |
| Gliclazide |  |  |  |  | An oral antihyperglycemic medication used in type 2 diabetes |
| Da Porto et al. (2020) | 60 mg | Once daily | NI |  |  |

***Note.*** The information listed corresponds to the comparator that was carried forward for the meta-analysis of this review, IBT = Intensive Behavioural Therapy, NI = no information

Supplementary Table 9. A table to show assessors’ judgement for the RoB 2.0 risk of bias for each domain and overall, for the primary outcome of each included study.

|  | **Domain one: randomisation process** | **Domain two: deviations from intended interventions** | **Domain three: missing outcome data** | **Domain four: measurement of the outcome** | **Domain five: selection of the reported result** | **Domain six: overall bias** |  |
| --- | --- | --- | --- | --- | --- | --- | --- |
| **Author (year)** | **Judgement** | **Judgement** | **Judgement** | **Judgement** | **Judgement** | **Judgement** |  |
| Allison et al. (2022) | High | High | High | Some concerns | Low | High |  |
| Blüher et al. (2024) | Low | Low | Some concerns | Some concerns | Some concerns | Some concerns |  |
| Blundell et al. (2017) | Some concerns | Low | Low | Some concerns | Low | Some concerns |  |
| Chao et al. (2019) | Some concerns | High | Low | High | Low | High |  |
| Da Porto et al. (2020) | Some concerns | High | Some concerns | High | Some concerns | High |  |
| Davies et al. (2015) | Low | Some concerns | High | Some concerns | Some concerns | High | |
| Dubé et al. (2020) | Low | Some concerns | Low | Some concerns | Some concerns | Some concerns | |
| Friedrichsen et al. (2020) | Low | Low | Low | Some concerns | Some concerns | Some concerns |  |
| Gabe et al. (2024) | Low | Low | Low | Some concerns | Some concerns | Some concerns | |
| Gatta-Cherifi et al. (2024) | Low | Low | Some concerns | Some concerns | Low | Some concerns | |
| Gibbons et al. (2020) | Some concerns | Low | Low | Some concerns | Low | Some concerns |  |
| Gudbergsen et al. (2021) | Low | Some concerns | High | Some concerns | Some concerns | High | |
| Jensen et al. (2022) | Low | Low | High | Some concerns | Low | High | |
| Jensterle et al. (2023) | Low | High | High | Some concerns | High | High | |
| Kanu et al. (2025) | Low | High | High | Some concerns | High | High | |
| Kanu et al. (2025) | Low | High | High | Some concerns | High | High | |
| Lau et al. (2021) | Low | Some concerns | Low | Some concerns | Low | Some concerns |  |
| Martin et al. (2024) | Low | Low | Some concerns | Some concerns | Low | Some concerns | |
| McElroy et al. (2024) | Low | High | High | Some concerns | Low | High |  |
| Patino et al. (2025) | Low | Some concerns | Some concerns | Some concerns | Some concerns | Some concerns | |
| Pi-Sunyer et al. (2015) | Low | Some concerns | High | Some concerns | Some concerns | High | |
| Robert et al. (2015) | Some concerns | High | High | High | Low | High |  |
| Snaith et al. (2025) | Low | Low | Low | Some concerns | Low | Some concerns | |
| Wadden et al. (2013) | Low | Some concerns | Some concerns | Some concerns | Low | Some concerns |  |
| Wharton et al. (2023) | Low | Low | Some concerns | Some concerns | Low | Some concerns | |

# **Supplementary Figures**

Supplementary Figure 1. Decision tree indicating strategies to address multiplicity in effect sizes induced by multiple sources.

***Note.*** Created in BioRender. Costantini, I. (2026) https://BioRender.com/ k349rpx. BMI = Body Mass Index; BED = Binge Eating Disorder, RCT = randomized controlled trial; ITT = intent to treat.

Supplementary Figure 2. Meta-regressions for continuous covariates on combined binge eating-related outcomes.

***Note.*** BMI = Body Mass Index; β = meta-regression coefficient (change in effect size per one-unit increase in moderator); k = number of studies. τ² quantifies between-study variance, and I² reflects the proportion of variability attributable to heterogeneity rather than sampling error.

Supplementary Figure 3. Subgroup analysis of baseline binge eating disorder on primary binge eating-related outcomes.

Supplementary Figure 4. Subgroup analysis of baseline diabetes on primary binge eating-related outcomes.

Supplementary Figure 5. Sensitivity analysis excluding studies at overall high risk of bias for the combined primary outcome

Supplementary Figure 6. Leave-one-out sensitivity analysis for the combined primary outcome

**Note.** g = Hedges’ g (standardised mean difference); 95% CI = 95% confidence interval; each point represents the pooled estimate with one study omitted; horizontal lines indicate 95% CIs; dashed red line = pooled estimate from the full model (all studies included).

Supplementary Figure 7. Robustness of the pooled estimate to different correlation values.

**Note.** g = Hedges’ g (standardised mean difference); 95% CI = 95% confidence interval; I² = percentage of total variability due to between-study heterogeneity; r = assumed correlation used to impute the SD of change scores; red dotted line = primary analysis estimate (r = 0.50).

**References**

1.	Page M, Higgins J, Sterne JAC. Chapter 13: Assessing risk of bias due to missing evidence in a meta-analysis . In: In: Higgins JP, Thomas J, Chandler J, Cumpston M, Li T, Page MJ, et al, editor(s). Cochrane Handbook for Systematic Reviews of Interventions version 6.5. . Cochrane; 2024.

2.	Higgins JP, Thomas J, Chandler J, Cumpston M, Li T, Page MJ. Chapter 6: Choosing effect measures and computing estimates of effect [last updated August 2023].  . In: Higgins JP, Li T, Deeks JJ, editors. Cochrane Handbook for Systematic Reviews of Interventions version 6.5. Cochrane, 2024. . 2024.

3.	Wharton S, Batterham RL, Bhatta M, Buscemi S, Christensen LN, Frias JP, et al. Two‐year effect of semaglutide 2.4 mg on control of eating in adults with overweight/obesity: STEP-5. Obesity. 2023 Mar 18;31(3):703–15. doi:10.1002/oby.23673

4.	Shi L, Lin L. The trim-and-fill method for publication bias: practical guidelines and recommendations based on a large database of meta-analyses. Medicine. 2019 Jun;98(23):e15987. doi:10.1097/MD.0000000000015987

5.	Gormally J, Black S, Daston S, Rardin D. The assessment of binge eating severity among obese persons. Addictive Behaviors. 1982 Jan;7(1):47–55. doi:10.1016/0306-4603(82)90024-7

6.	Fairburn CG, Beglin SJ. Assessment of eating disorders: interview or self-report questionnaire? Int J Eat Disord. 1994 Dec;16(4):363–70. PubMed PMID: 7866415.

7.	Cappelleri JC, Bushmakin AG, Gerber RA, Leidy NK, Sexton CC, Lowe MR, et al. Psychometric analysis of the Three-Factor Eating Questionnaire-R21: results from a large diverse sample of obese and non-obese participants. Int J Obes. 2009 Jun 28;33(6):611–20. doi:10.1038/ijo.2009.74

8.	Dalton M, Finlayson G, Hill A, Blundell J. Preliminary validation and principal components analysis of the Control of Eating Questionnaire (CoEQ) for the experience of food craving. Eur J Clin Nutr. 2015 Dec 8;69(12):1313–7. doi:10.1038/ejcn.2015.57

9.	Stunkard AJ, Messick S. The three-factor eating questionnaire to measure dietary restraint, disinhibition and hunger. J Psychosom Res. 1985 Jan;29(1):71–83. doi:10.1016/0022-3999(85)90010-8

10.	Stunkard AJ, Messick S. Eating Inventory. PsycTESTS Dataset. 2012. doi:10.1037/t15085-000

11.	Allison KC, Chao AM, Bruzas MB, McCuen‐Wurst C, Jones E, McAllister C, et al. A pilot randomized controlled trial of liraglutide 3.0 mg for binge eating disorder. Obes Sci Pract. 2023 Apr 26;9(2):127–36. doi:10.1002/osp4.619

12.	Chao AM, Wadden TA, Walsh OA, Gruber KA, Alamuddin N, Berkowitz RI, et al. Effects of Liraglutide and Behavioral Weight Loss on Food Cravings, Eating Behaviors, and Eating Disorder Psychopathology. Obesity. 2019 Dec 20;27(12):2005–10. doi:10.1002/oby.22653

13.	Da Porto A, Casarsa V, Colussi G, Catena C, Cavarape A, Sechi L. Dulaglutide reduces binge episodes in type 2 diabetic patients with binge eating disorder: A pilot study. Diabetes & Metabolic Syndrome: Clinical Research & Reviews. 2020 Jul;14(4):289–92. doi:10.1016/j.dsx.2020.03.009

14.	Davies MJ, Bergenstal R, Bode B, Kushner RF, Lewin A, Skjøth TV, et al. Efficacy of Liraglutide for Weight Loss Among Patients With Type 2 Diabetes. JAMA. 2015 Aug 18;314(7):687. doi:10.1001/jama.2015.9676

15.	Gudbergsen H, Overgaard A, Henriksen M, Wæhrens EE, Bliddal H, Christensen R, et al. Liraglutide after diet-induced weight loss for pain and weight control in knee osteoarthritis: a randomized controlled trial. Am J Clin Nutr. 2021 Feb;113(2):314–23. doi:10.1093/ajcn/nqaa328

16.	McElroy SL, Guerdjikova AI, Blom TJ, Mori N, Romo-Nava F. Liraglutide in Obese or Overweight Individuals With Stable Bipolar Disorder. J Clin Psychopharmacol. 2024 Mar;44(2):89–95. doi:10.1097/JCP.0000000000001803

17.	Pi-Sunyer X, Astrup A, Fujioka K, Greenway F, Halpern A, Krempf M, et al. A Randomized, Controlled Trial of 3.0 mg of Liraglutide in Weight Management. New England Journal of Medicine. 2015 Jul 2;373(1):11–22. doi:10.1056/NEJMoa1411892

18.	Robert SA, Rohana AG, Shah SA, Chinna K, Wan Mohamud WN, Kamaruddin NA. Improvement in binge eating in non-diabetic obese individuals after 3 months of treatment with liraglutide – A pilot study. Obes Res Clin Pract. 2015 May;9(3):301–4. doi:10.1016/j.orcp.2015.03.005

19.	Wadden T. https://clinicaltrials.gov/study/NCT00781937 [Internet]. 2017 [cited 2025 Oct 20]. Comparison of Liraglutide Versus Placebo in Weight Loss Maintenance in Obese Subjects: SCALE - Maintenance. Available from: https://clinicaltrials.gov/study/NCT00781937

20.	Kanu C, Boye KS, Poon JL, Goetz I, Williamson S, Lou J, et al. Appetite, eating attitudes, and eating behaviours during treatment with retatrutide in adults with type 2 diabetes: Results of a phase 2 study. Diabetes Obes Metab. 2025 Sep 8. doi:10.1111/dom.70097

21.	Gatta-Cherifi B, Mohammedi K, Cariou T, Poitou C, Touraine P, Raverot G, et al. Impact of exenatide on weight loss and eating behavior in adults with craniopharyngioma-related obesity: the CRANIOEXE randomized placebo-controlled trial. Eur J Endocrinol. 2024 Mar 30;190(4):257–65. doi:10.1093/ejendo/lvae024

22.	Snaith JR, Frampton R, Samocha-Bonet D, Greenfield JR. Tirzepatide in Adults With Type 1 Diabetes: A Phase 2 Randomized Placebo-Controlled Clinical Trial. Diabetes Care. 2026 Jan 1;49(1):161–70. doi:10.2337/dc25-2379

23.	Patino LR, Strawn JR, Adler CM, Blom TJ, Welge JA, DelBello MP. A double-blind, placebo-controlled trial of exenatide for the treatment of olanzapine-related weight gain in obese and overweight adults. J Affect Disord. 2025 Aug;382:116–22. doi:10.1016/j.jad.2025.04.046

24.	Blüher M, Rosenstock J, Hoefler J, Manuel R, Hennige AM. Dose–response effects on HbA1c and bodyweight reduction of survodutide, a dual glucagon/GLP-1 receptor agonist, compared with placebo and open-label semaglutide in people with type 2 diabetes: a randomised clinical trial. Diabetologia. 2024 Mar 14;67(3):470–82. doi:10.1007/s00125-023-06053-9

25.	Lau DCW, Erichsen L, Francisco AM, Satylganova A, le Roux CW, McGowan B, et al. Once-weekly cagrilintide for weight management in people with overweight and obesity: a multicentre, randomised, double-blind, placebo-controlled and active-controlled, dose-finding phase 2 trial. The Lancet. 2021 Dec;398(10317):2160–72. doi:10.1016/S0140-6736(21)01751-7

26.	Blundell J, Finlayson G, Axelsen M, Flint A, Gibbons C, Kvist T, et al. Effects of once‐weekly semaglutide on appetite, energy intake, control of eating, food preference and body weight in subjects with obesity. Diabetes Obes Metab. 2017 Sep 5;19(9):1242–51. doi:10.1111/dom.12932

27.	Friedrichsen M, Breitschaft A, Tadayon S, Wizert A, Skovgaard D. The effect of semaglutide 2.4 mg once weekly on energy intake, appetite, control of eating, and gastric emptying in adults with obesity. Diabetes Obes Metab. 2021 Mar 3;23(3):754–62. doi:10.1111/dom.14280

28.	Gabe MBN, Breitschaft A, Knop FK, Hansen MR, Kirkeby K, Rathor N, et al. Effect of oral semaglutide on energy intake, appetite, control of eating and gastric emptying in adults living with obesity: A randomized controlled trial. Diabetes Obes Metab. 2024 Oct 31;26(10):4480–9. doi:10.1111/dom.15802

29.	Gibbons C, Blundell J, Tetens Hoff S, Dahl K, Bauer R, Bækdal T. Effects of oral semaglutide on energy intake, food preference, appetite, control of eating and body weight in subjects with type 2 diabetes. Diabetes Obes Metab. 2021 Feb 27;23(2):581–8. doi:10.1111/dom.14255

30.	Kanu C, Wu Q, Poon JL, Goetz I, Perez‐Nieves M, Boye K, et al. Association between patient‐reported eating behaviours and weight change: Secondary analyses of a randomized, double‐blind trial comparing retatrutide and placebo in people with obesity or overweight. Diabetes Obes Metab. 2025 Oct 30;27(10):6088–91. doi:10.1111/dom.16662

31.	Martin CK, Carmichael OT, Carnell S, Considine R V., Kareken DA, Dydak U, et al. Tirzepatide on ingestive behavior in adults with overweight or obesity: a randomized 6-week phase 1 trial. Nat Med. 2025 Sep 24;31(9):3141–50. doi:10.1038/s41591-025-03774-9

32.	Jensen SBK, Janus C, Lundgren JR, Juhl CR, Sandsdal RM, Olsen LM, et al. Exploratory analysis of eating- and physical activity-related outcomes from a randomized controlled trial for weight loss maintenance with exercise and liraglutide single or combination treatment. Nat Commun. 2022 Aug 15;13(1):4770. doi:10.1038/s41467-022-32307-y

33.	Dubé M, D’Amours M, Weisnagel SJ. Effect of liraglutide on food consumption, appetite sensations and eating behaviours in overweight people with type 1 diabetes. Diabetes Obes Metab. 2020 Aug 6;22(8):1417–24. doi:10.1111/dom.14050

34.	Jensterle M, Ferjan S, Battelino T, Kovač J, Battelino S, Šuput D, et al. Does intervention with GLP-1 receptor agonist semaglutide modulate perception of sweet taste in women with obesity: study protocol of a randomized, single-blinded, placebo-controlled clinical trial. Trials. 2021 Dec 19;22(1):464. doi:10.1186/s13063-021-05442-y

35.	Karlsson J, Persson LO, Sjöström L, Sullivan M. Psychometric properties and factor structure of the Three-Factor Eating Questionnaire (TFEQ) in obese men and women. Results from the Swedish Obese Subjects (SOS) study. Int J Obes. 2000 Dec 4;24(12):1715–25. doi:10.1038/sj.ijo.0801442

36.	Bryant EJ, Rehman J, Pepper LB, Walters ER. Obesity and Eating Disturbance: the Role of TFEQ Restraint and Disinhibition. Curr Obes Rep. 2019 Dec 7;8(4):363–72. doi:10.1007/s13679-019-00365-x

37.	Lowe MR, Caputo GC. Binge eating in obesity: Toward the specification of predictors. International Journal of Eating Disorders. 1991 Jan;10(1):49–55. doi:10.1002/1098-108X(199101)10:1<49::AID-EAT2260100106>3.0.CO;2-X

38.	Stunkard AJ, Messick S. The three-factor eating questionnaire to measure dietary restraint, disinhibition and hunger. J Psychosom Res. 1985 Jan;29(1):71–83. doi:10.1016/0022-3999(85)90010-8

39.	Berg KC, Peterson CB, Frazier P, Crow SJ. Psychometric evaluation of the eating disorder examination and eating disorder examination-questionnaire: a systematic review of the literature. Int J Eat Disord. 2012 Apr;45(3):428–38. doi:10.1002/eat.20931 PubMed PMID: 21744375.

40.	Trolio V, Hales EL, Biçaker E, Miller AE, Racine SE. Cognitive restraint versus restriction: An investigation of daily eating patterns among women with eating disorders characterized by restriction. Appetite. 2026 Jul;222:108502. doi:10.1016/j.appet.2026.108502

41.	Sultson H, Kukk K, Akkermann K. Positive and negative emotional eating have different associations with overeating and binge eating: Construction and validation of the Positive-Negative Emotional Eating Scale. Appetite. 2017 Sep;116:423–30. doi:10.1016/j.appet.2017.05.035

42.	Latner JD, Mond JM, Kelly MC, Haynes SN, Hay PJ. The loss of control over eating scale: Development and psychometric evaluation. International Journal of Eating Disorders. 2014 Sep 26;47(6):647–59. doi:10.1002/eat.22296

43.	Yanovski SZ, Marcus MD, Wadden TA, Walsh BT. The Questionnaire on Eating and Weight Patterns‐5: An updated screening instrument for binge eating disorder. International Journal of Eating Disorders. 2015 Apr 26;48(3):259–61. doi:10.1002/eat.22372

44.	van Strien T, Frijters JER, Bergers GPA, Defares PB. The Dutch Eating Behavior Questionnaire (DEBQ) for assessment of restrained, emotional, and external eating behavior. International Journal of Eating Disorders. 1986 Feb;5(2):295–315. doi:10.1002/1098-108X(198602)5:2<295::AID-EAT2260050209>3.0.CO;2-T

45.	Gormally J, Black S, Daston S, Rardin D. The assessment of binge eating severity among obese persons. Addictive Behaviors. 1982 Jan;7(1):47–55. doi:10.1016/0306-4603(82)90024-7

46.	Schmiedel O, Ivey M, Liu A, Murphy R. The New Zealand eating behavior questionnaire – Validation study for a novel assessment tool to describe actionable eating behavior traits. Appetite. 2023 Dec;191:107066. doi:10.1016/j.appet.2023.107066

47.	Papini NM, Jung M, Cook A, Lopez N V., Ptomey LT, Herrmann SD, et al. Psychometric properties of the 26-item eating attitudes test (EAT-26): an application of rasch analysis. J Eat Disord. 2022 May 4;10(1):62. doi:10.1186/s40337-022-00580-3

48.	Burton AL, Abbott MJ. The revised short-form of the Eating Beliefs Questionnaire: Measuring positive, negative, and permissive beliefs about binge eating. J Eat Disord. 2018 Dec 6;6(1):37. doi:10.1186/s40337-018-0224-0
